# The Effect of Addition of Different Amounts of Chitosan Powder to the Batter and Chitosan Coating of Tea Sausage (Dry-Fermented Sausage) on the Physico-Chemical, Sensory and Microbiological Quality

**DOI:** 10.3390/foods15173125

**Published:** 2026-09-02

**Authors:** Jelena Latinović, Vladimir Tomović, Lato Pezo, Senka Popović, Nevena Hromiš, Sunčica Kocić-Tanackov, Aleksandra Martinović, Dragan Vujadinović, Vesna Đorđević

**Affiliations:** 1 Faculty of Technology Novi Sad, University of Novi Sad, 21000 Novi Sad, Serbia; jelenarlatinovic@gmail.com (J.L.); madjarev@uns.ac.rs (S.P.); nevena.krkic@uns.ac.rs (N.H.); suncicat@uns.ac.rs (S.K.-T.); 2 Ribella Foods Doo, 21234 Bački Jarak, Serbia; 3 Institute of General and Physical Chemistry, University of Belgrade, 11158 Belgrade, Serbia; latopezo@yahoo.co.uk; 4 Faculty for Food Technology, Food Safety and Ecology, University of Donja Gorica, Donja Gorica, 81000 Podgorica, Montenegro; aleksandra.martinovic@udg.edu.me; 5 Faculty of Technology Zvornik, University of East Sarajevo, 75400 Zvornik, Bosnia and Herzegovina; dragan.vujadinovic@tfzv.ues.rs.ba; 6 Institute of Meat Hygiene and Technology, 11040 Belgrade, Serbia; vesna.djordjevic@inmes.rs

**Keywords:** chitosan, tea sausage, dry-fermented sausage, quality

## Abstract

The effect of chitosan addition at amounts of 0, 0.25 and 0.50% and chitosan coating on pH, instrumental surface and cut cross-section colour (L*—lightness, a*—redness, b*—yellowness, *h*—hue angle, C*—chroma-saturation index and λ—dominant wavelength) and texture (hardness, springiness, cohesiveness and chewiness) parameters, and overall sensory and microbiological (TPC—total plate count and LAB—lactic acid bacteria) quality of dry-fermented sausages (tea sausage) were investigated. The addition of chitosan increased the pH, surface λ and cut cross-section *h* (0.50%), and decreased surface b*, C* (0.50%) and *h*, and cut cross-section a* (0.50%) and λ (0.50%), while chitosan coating increased surface L* and *h*, and decreased surface a*, b*, C* and λ, and cut cross-section L*. Hardness (0.50%), springiness, cohesiveness and chewiness were lower in the sausages with chitosan. The chitosan coating also led to a decrease in hardness, but at the same time increased springiness and cohesiveness. The chitosan addition improved the overall sensory quality, while the chitosan coating had the opposite trend. The microbiological results indicated that addition of chitosan had reduced TPC (0.50%), while chitosan coating had reduced LAB. In addition, the adding of chitosan did not affect surface L* and a*, cut cross-section L*, b* and C*, and LAB, while pH, cut cross-section a*, b*, C*, *h* and λ, chewiness and TPC were not affected by the chitosan coating. The two-way interaction between amount of chitosan and the chitosan coating had a significant (*p* = 0.038–<0.001) effect on the pH, surface a*, *h* and λ, cut cross-section L*, a*, C*, *h* and λ, hardness and overall sensory and microbiological (TPC and LAB) quality. In practice, chitosan can be applied as a food additive to control and improve the quality of dry-fermented pork sausages.

## 1. Introduction

Tea sausage (“Čajna kobasica”) is the most popular and the most eaten dry-fermented sausage in Serbia (personal data obtained from the meat industry). In Serbia, the name ’’tea sausage’’ is said to derive from the habit of serving sandwiches made with this sausage at teatime. Tea sausage is typically produced from pork meat, pork fat, salt, and a mixture of spices, with nitrite salt and other ingredients being used depending on the formulation and production practices. The sausage undergoes fermentation, drying, and ripening, during which physico-chemical and microbiological changes contribute to the development of its characteristic odour, taste, texture, and colour. Compared with other dry-fermented sausages, tea sausage is characterized by its specific formulation, relatively fine particle size, distinctive sensory properties, and production practices associated with Serbian meat-processing traditions. Its quality and shelf stability are influenced by factors such as formulation, fermentation and drying conditions, packaging, and storage conditions.

The basic parameter that regulates the chemical quality of tea sausage (dry-fermented sausage) in Serbia is the meat protein content, being at least 20%, without the addition of other animal or vegetable proteins. Also, dry-fermented sausages must contain less than 35% water and final pH must reach 5.0 [1].

Production of this dry-fermented and cold-smoked sausage is one of meat-processing areas that occupy the interest of scientists in Serbia in the last several decades [2,3,4,5,6,7,8,9,10].

Chitosan is a naturally derived biopolymer obtained primarily through the partial deacetylation of chitin, which is abundantly found in the exoskeletons of crustaceans and insects and in the cell walls of fungi [11]. Over the past few decades, it has attracted increasing attention across a range of industries—including biomedicine, textiles, pharmaceuticals, cosmetics, and, most notably, the food industry. Its antimicrobial, antioxidant and film-forming abilities reinforced by the polymer’s biocompatible, biodegradable and nontoxic nature have fostered its use in food packaging and preservation [12,13,14,15,16,17,18]. Numerous studies have investigated the use of chitosan as an additive or as an edible film and coating, highlighting its suitability as a packaging material for meat products. A recent comprehensive review by Fernando et al. [13] detailed the potential applications of chitosan in meat and meat-based products. Their overall conclusion emphasized that incorporating chitosan in the form of films, coatings, or additives contributes to shelf-life extension and positively influences sensory attributes. Although the application of chitosan in fermented and dry-fermented sausages has been extensively investigated, most previous studies have focused on individual aspects of chitosan application, such as its antimicrobial activity, effects on physico-chemical properties, or its use as an edible coating. However, limited information is available on the combined effects of different chitosan concentrations, chitosan coatings, packaging systems, and prolonged storage on the overall quality of tea sausage. In particular, the simultaneous application of chitosan incorporated into the sausage batter and chitosan coating represents an important aspect of the novelty of the present study, as this combined approach has received limited attention in previous research. Furthermore, the interaction between chitosan treatment and packaging conditions, including vacuum packaging and modified atmosphere packaging (MAP), during extended storage has not been sufficiently investigated for this specific type of Serbian dry-fermented sausage. Therefore, the present study was designed to evaluate the effects of chitosan incorporation into the sausage batter and the application of chitosan coating, in combination with different packaging systems and storage periods, on the physico-chemical, textural, microbiological, and sensory properties of tea sausage. The study provides additional information on the potential of combining chitosan incorporation and surface coating as complementary technological approaches for maintaining product quality and extending the shelf life of dry-fermented sausages. The findings may be relevant to the meat industry for the development of strategies aimed at improving product stability and quality during storage while maintaining the characteristic properties of fermented meat products.

This study investigates the effects of incorporating different concentrations of chitosan into the sausage batter and applying a chitosan coating to tea sausage on its physico-chemical properties (pH, instrumental colour and texture), overall sensory quality and microbiological characteristics (total plate count and lactic acid bacteria) during storage under vacuum and modified atmosphere packaging.

## 2. Materials and Methods

### 2.1. Coating Preparation

The procedure for chitosan (Sigma-Aldrich Chemical, St. Louis, MO, USA) coating preparation was described in detail in Krkić et al. [17,18]. Chitosan obtained from crab shells (Chitosan from crab shells, highly viscous; Sigma-Aldrich, product No. 48165, CAS No. 9012-76-4) was used in the present study. The chitosan was supplied as a white to faint beige powder. According to the manufacturer’s specifications, the content of insoluble matter was ≤1%, the ignition residue was ≤2% (as sulphate), and the loss on drying was ≤12%. The viscosity was >400 mPa·s, determined for a 1% solution in acetic acid at 20 °C. The chitosan coating was prepared by dissolving chitosan powder in 1% acetic acid to reach a chitosan mass per volume ratio of 4 kg·m^−3^. The solution was stirred overnight with a magnetic stirrer in order to dissolve the chitosan.

### 2.2. Sausage Preparation

Dry-fermented sausages were made from pork shoulder meat and pork fat (firm subcutaneous fat—backfat) in the ratio of 80:20. Three types of tea sausages (50 kg each) were manufactured in a single batch per treatment group, containing 0% (Ch0/B—assigned as control sausages), 0.25% (Ch0.25/B—assigned as medium-chitosan sausages) and 0.50% (Ch0.50/B—assigned as high-chitosan sausages) chitosan powder, respectively. Repeated measurements were performed within each batch to assess variability.

Backfat and a portion of the pork shoulder were frozen at −25 °C and then processed using a bowl cutter (TTChop55V, Tipper Tie Alpina GmbH, Gossau, Sankt Gallen, Switzerland) before the addition of pre-minced pork shoulder (3–5 mm diameter plate), nitrite salt (2.6%), spices and their extracts (white pepper powder, black pepper extract, allspice extract (*Pimenta dioica*) and nutmeg extract, 0.25%), Mix 63-0 Top Start (enhancer for the growth of microorganisms, dextrose, sucrose, antioxidant—E300, 0.55%) and a starter culture mixture (*Micrococcus*/*Lactobacillus*/*Pediococcus*, 0.015%, Lallemand Specialty Cultures, La Ferté-sous-Jouarre, France). The batter was mixed for 3 min and stuffed into 37 mm diameter collagen casings (Cutisin s.r.o, Jilemnice, Czech Republic) using vacuum filler VF622 (Handtmann, Biberachan der Riss, Germany), making around 100 sausages of approximately 33 cm in length. For each treatment, the required amount of chitosan was accurately weighed (125 g for the 0.25% treatment and 250 g for the 0.50% treatment). To ensure uniform distribution, the chitosan was first thoroughly mixed with a smaller portion of the meat batter to obtain a homogeneous premix. The premix was then gradually incorporated into the remaining meat batter, followed by thorough mixing until a uniform distribution of chitosan throughout the batter was achieved. After being left to rest overnight, the sausages were transferred to a smoking/fermentation chamber (Maurer Söhne GmbH und Co. KG, Munich, Germany), where they were cold smoked for first 5 days (3 × 3 h per day) using beech chips (Supplementary Material, Table S1). After 5 days, the sausages were transferred to a drying/ripening chamber (Maurer Söhne GmbH und Co. KG, Munich, Germany), where they were kept until the end of the drying [6]. The temperature, relative humidity and air velocity conditions in the smoking/fermentation and drying/ripening chamber are presented in Table S1 (Supplementary Material). During the sausage production process, the temperature was gradually reduced from 20 to 15 °C, while the relative humidity was gradually decreased from 90 to 70%.

Following the drying process, half of the sausages were coated with three layers of a coating solution using a sponge brush (designated as coated sausages—Co). Each layer was allowed to dry overnight before the subsequent layer was applied. The remaining sausages were left uncoated (designated as uncoated sausages—UCo) [17,18].

After coating, the coated sausages were divided into 2 groups [Vacuum (multilayer heat-shrinkable high-barrier bag based on EVOH) packaging, assigned as vacuum-packaged sausages—V; and MAP (top and bottom foil: Ecoweb M/Pap 72, PET/PE/EVOH/PE and Ecopet V 300, APET/EVOH/LDPE, Südpack, Verpackungen SE & Co., Ochsenhausen, Germany (70% nitrogen and 30% carbon dioxide)) packaging, assigned as modified-atmosphere-packaged sausages—MAP] and then packaged by using a Multivac C500 vacuum chamber (Multivac Sepp Heggenmuller Gmbh & Co, Wolfertschwenden, Germany) and a packaging machine for MAP (Tiromat CFS Compact M420, Biedenkopf, Germany). The total number of different groups of manufactured sausages was 27 (3 + 3 × 2 × 2 × 2) (Supplementary Material, Table S2). After packaging, the packaged sausages were stored at 15 °C [1].

Samples for analysis were taken at day 0 (meat/fat mixture immediately after stuffing, assigned as B) and day 21 (end of drying process, assigned as 0), and after 45 (assigned as 45) and 90 days (assigned as 90) of storage under defined conditions, i.e., 66 and 111 days of stuffing production. At each sampling time, 10 sausages per group were analysed.

### 2.3. Physico-Chemical Analysis

The pH [19] of the samples was determined using a digital pH meter Testo 205 (Testo, Sparta, NJ, USA), which uses automatic temperature compensation. Before measurement it was calibrated using standard buffers (pH = 4.00 ± 0.05 and pH = 7.00 ± 0.01 at 20 ± 2 °C). pH values were measured for 3 batters (5 readings per batter in aggregate samples) and for 10 sausages from each group of dry-fermented sausages (15 readings per group of sausage).

The moisture, protein (nitrogen × 6.25), total fat, total ash, chloride, total phosphorus and nitrite contents were determined according to International Organization for Standardization (ISO) procedures [20,21,22,23,24,25,26]. Chemical composition was determined in the aggregate samples of batters and sausages.

The instrumental parameters of surface and fresh cut cross-section CIE [27] colour expressed as lightness—L*, redness—a*, yellowness—b*, chroma-saturation index—C* [C* = (a*^2^ + b*^2^)^1/2^], hue angle—*h* [*h* = arctangent (b*/a*)] and dominant wavelength—λ (nm) of the samples were measured immediately after slicing. Colour parameters were determined using a Minolta Chroma Meter CR-400 (Minolta Co., Ltd., Osaka, Japan) using D-65 lighting, a 2° standard observer angle and an 8 mm aperture in the measuring head. Prior to measurement it was calibrated using a Minolta calibration plate (No. 11333090; Y = 92.9, x = 0.3159; y = 0.3322). Colour parameters were measured on 10 sausages from each experimental group. For each sausage, three measurements were taken at different locations to account for potential variability in colour distribution. Thus, a total of 30 measurements were obtained per experimental group.

The instrumental texture profile analysis (TPA)—including hardness (g), springiness, cohesiveness and chewiness (g)—was conducted following the detailed procedure outlined by Tomović et al. [8]. TPA measurements were also performed on 10 sausages per group, resulting in 30 readings for each group.

### 2.4. Sensory Analyses

Sensory evaluation was conducted by a trained panel of ten members, aged between 25 and 50, across one session [8]. Panel training followed the guidelines outlined in ISO 8586 [28], and the evaluations were performed in a sensory laboratory designed in accordance with ISO 8589 [29]. The sensory analysis was conducted in the accredited sensory testing laboratory of the Institute of Meat Hygiene and Technology. Before evaluation, sausage samples were allowed to equilibrate to room temperature for approximately 30 min and were labelled with random three-digit codes. Each sausage was sliced into 2.5 mm thick pieces and presented on white porcelain plates [8]. The overall sensory quality was evaluated based on five pre-selected attributes: external appearance, cross-sectional appearance, cross-sectional colour, odour and taste, and oral texture.

Sensory evaluation was performed using five sensory attributes: external appearance, cross-sectional appearance, cross-sectional colour, odour and taste, and oral texture. Each attribute was assigned a coefficient of importance (CI), reflecting its relative contribution to the overall quality of the sausage. The CI values for the five attributes were 2, 5, 3, 7, and 3, respectively. Thus, odour and taste, which were considered to have the greatest influence on overall sensory quality, received the highest coefficient (CI = 7), whereas external appearance received the lowest coefficient (CI = 2). Sensory characteristics were initially evaluated using a 5-point categorical scale, with scores ranging from 1 to 5, where 5 represented the highest sensory quality and 1 the lowest. To allow more precise differentiation between samples, each integer category was further divided into four equal intervals, resulting in a 20-point refined scale. Panellists assigned scores according to the intensity of the observed positive characteristics or sensory defects. The scoring procedure was based on deducting points from the maximum score of 5 according to the intensity of the observed defects, following the internal laboratory protocol used for the sensory evaluation of dry-fermented sausages [30]. All panellists evaluated samples from all experimental treatments, using the same scoring criteria and evaluation procedure. For each panellist, the overall sensory quality score was calculated by weighting the score assigned to each attribute according to its coefficient of importance. The weighted scores were summed and divided by the sum of all CI values according to the following equation:

Overall sensory quality score = Σ (attribute score × CI)/Σ CI

The sum of the CI values was 20. Therefore, attributes with higher CI values contributed proportionally more to the final overall quality score. This approach allowed the overall sensory quality to reflect the relative importance of each individual sensory attribute.

### 2.5. Microbiological Analysis

The microbiological quality [total plate count (TPC), lactic acid bacteria (LAB), *Enterobacteriaceae*, *Escherichia coli*, *Salmonella* spp., *Listeria monocytogenes*, sulphite-reducing clostridia count and yeasts and moulds] was determined according to International Organization for Standardization (ISO) procedures [31,32,33,34,35,36,37,38]. Microbiological quality attributes were measured for 3 batters (5 readings per batter in aggregate samples) and for 10 sausages from each group of dry-fermented sausages (10 readings per group of sausage). The results were expressed as a log number of colony forming units per gram (log CFU/g).

### 2.6. Statistical Analysis

Data were analysed using a mixed-effects factorial ANOVA model. Chitosan concentration (0, 0.25, and 0.50%), chitosan coating (with or without coating), packaging type (vacuum or MAP), and storage time (0, 45, and 90 days) were included as fixed effects, while experimental repetition was treated as a random effect. Chemical composition analyses were performed on a composite sample prepared from the three independent sausage batches. The updated statistical model was structured to reflect the experimental design accurately, incorporating both fixed and random effects. Fixed factors included the amount of chitosan, chitosan coating, packaging type and storage time. The repetitions were treated as a random effect to account for variability between experimental runs. The two-way interactions among fixed and random effects were also included to examine potential synergistic or antagonistic effects between factors.

The main effects (amount of chitosan, chitosan coating, packaging and storage day) were also evaluated in a separate ANOVA study. All data were expressed as mean values with their standard errors. The two-way, three-way and four-way interactions between these effects were also tested. Differences among treatment means were compared using Tukey’s HSD test (*p* < 0.05). Principal component analysis (PCA) was employed to elucidate and identify patterns within the collected data. The statistical software TIBCO Statistica, version 14.0.0.15 [39] was used for data analysis.

Separate compositional analyses of each meat and fat batch were not performed. The chemical composition data were obtained as a general characterization of the raw materials used in the study and do not represent batch-specific replicated measurements. Accordingly, these results are presented for descriptive purposes and not for statistical comparison across experimental repetitions.

## 3. Results and Discussion

### 3.1. Physico-Chemical and Microbiological Characteristics of Tea Sausage Batters

The moisture, protein, total fat, total ash, chloride, total phosphorus and nitrite contents, as well as the microbiological quality (TPC and LAB), of all three tea sausage batters (Ch0/B, Ch0.25/B and Ch0.50/B) are presented in Table 1.

In this study, the pH value, TPC and LAB of batter were significantly affected (*p* < 0.05) by the addition of chitosan. The pH values were the highest for the Ch0.50/B batter (6.45; *p* < 0.05), while Ch0/B batter had the lowest pH values (6.01; *p* < 0.05) (Table 1). Likewise, several authors found same effect of chitosan on the pH of minced/ground beef meat with chitosan [40,41,42], pork model burgers with chitosan [43], beef burgers with chitosan [44], fresh pork sausages with chitosan [45,46], chitosan-coated Harbin red sausage [47], vacuum-packed cooked pork sausages—emulsion-type pork sausages with chitosan [48], cooked turkey sausages with chitosan [49] or fermented cooked sausages with chitosan [50]. The pH increase observed in this study, as well as in other research, is attributed to the alkaline nature of chitosan. This finding aligns with previous reports indicating that the pH of chitosan solutions typically ranges from 7.2 to 7.8 [42,48]. In contrast, the use of chitosan did not influence pH values ([51], chitosan-coated dry-fermented sausages; [52], minced beef meat with chitosan; [53], vacuum-packed cooked pork sausages—emulsion-type pork sausages with chitosan; [54], Ham Visking—a type of cooked pork sausages with chitosan; [14], chitosan-coated dry-fermented sausages—*Petrovská klobása*; [55], fresh pork sausages with chitosan; [56], chitosan-coated frankfurter-type sausages) or resulted in a pH decrease ([57], frankfurter-type sausages with chitosan; [51,58], Chinese-style sausage with chitosan; [59], cooked chicken sausage with chitosan).

TPC and LAB values were the highest for the Ch0/B batter (4.30 and 5.99, respectively; *p* < 0.05), while Ch0.25/B batter had the lowest TPC and LAB values (3.99 and 5.04; *p* < 0.05) (Table 1). Thus, in the fresh sausage batter, the Ch0.25/B treatment resulted in significantly lower TPC and LAB counts compared with both the control (Ch0/B) and Ch0.50/B treatments (*p* < 0.05). Although chitosan has been confirmed as attractive biomacromolecules with relevant antimicrobial properties ([40,41,43,45,46,47,50,52,54,55,57,59,60], chitosan-coated dry-cured meat products; [61], cooked chicken meat products with chitosan; [62], chitosan-coated cooked beef/chicken sausages; [63], fermented meat sausages with chitosan; [64], fresh pork sausages with chitosan; [65], chitosan-coated ground meat; [66], dry-fermented sausages—Turkish sausage (sucuk) with chitosan; [67], chitosan-coated ready-to-cook meat products; [68], cured chicken meat with chitosan), the present results suggest that chitosan had an inhibitory effect on both TPC and LAB in the fresh sausage batter. Interestingly, the lowest microbial counts were observed at the intermediate chitosan concentration (0.25%), rather than at the higher concentration (0.50%), indicating that the observed response was not directly proportional to the chitosan concentration. The antimicrobial activity of chitosan in complex food matrices may depend on several factors, including its concentration, interactions with meat components, pH, physico-chemical characteristics of the matrix, and the physiological state of the microorganisms. Therefore, the significantly lower TPC and LAB counts observed at 0.25% chitosan may reflect a concentration-specific response resulting from the complex interaction between chitosan and the meat matrix rather than a simple dose-dependent effect. This finding was observed in the fresh sausage batter and should not be generalized to the subsequent fermentation and storage stages. Contrarily, other researchers found that the use of chitosan did not affect the microbial population in vacuum-packed cooked pork sausages—emulsion-type pork sausages [48,53] and chitosan-coated dry-fermented sausages [50]. No foodborne pathogens (*Enterobacteriaceae*, *Escherichia coli*, *Salmonella* spp., *Listeria monocytogenes* and sulphite-reducing clostridia) and yeasts and moulds were detected in any batter or tea sausage samples during the storage period (90 days).

### 3.2. ANOVA of Physico-Chemical, Sensory and Microbiological Characteristics: Role of Random and Fixed Factors

Although repetitions (RP) were initially included in the model as a random factor, preliminary analyses revealed that this term had minimal influence on the variability of the measured traits. RP was found to be statistically significant in the sensory analysis (*p* < 0.001), indicating that variability between repetitions plays an important role in influencing sensory evaluation results. Moreover, several interactions involving RP—specifically RP × ST (*p* < 0.001) and RP × CC (*p* < 0.05)—also show significant effects, suggesting that the influence of storage time and chitosan coating on sensory attributes varies across repetitions. In contrast, interactions such as RP × AC and RP × P were not significant. These findings emphasize that the random variability captured by repetitions and its interactions with certain fixed factors play a critical role in shaping the sensory analysis outcomes. However, RP was excluded from the analysis of instrumental data due to its lack of statistical significance. Detail analysis is presented in the Supplementary Material (Tables S3–S8).

Consequently, to further investigate the effects of the fixed factors alone, a separate analysis of variance (ANOVA) was conducted considering only the fixed effects: amount of chitosan (AC), chitosan coating (CC), packaging (P) and storage time (ST), along with their relevant interactions. Where significant effects were detected, mean comparisons were performed using Tukey’s honestly significant difference (HSD) test to identify statistically distinct treatment groups (*p* < 0.05).

### 3.3. Physico-Chemical Characteristics of Tea Sausages

The moisture, protein, total fat, total ash, chloride, total phosphorus and nitrite contents of all three tea sausages at the end of the drying process (Ch0/0, Ch0.25/0 and Ch0.50/0) are presented in Table 2. The chemical analysis shows that all the tea sausages meet the requirements in terms of chemical composition [1] and level of additives [69].

Many significant (*p* < 0.05) or numerical differences were found in the mean values of the physico-chemical characteristics among different groups of tea sausages (data shown in the Supplementary Material, Tables S3–S15, Figures S1–S20).

Mean pH values (*p* < 0.05) in all 27 tea sausage groups varied between 5.24 (Ch0/Co/MAP/90) and 5.67 (Ch0.50/UCo/V/45) (Supplementary Material, Figure S1). It was in strong agreement with results of our previous studies [7,8,9]. Chitosan coating and storage time had no significant effect (*p* > 0.05) on the pH values, while the amount of chitosan and packaging had significant effect (*p* < 0.05) on the pH values. Tea sausages produced with 0% chitosan had the lowest pH values, while tea sausages produced with 0.50% chitosan had the highest pH values (Table 3), which was consistent with the pH values obtained in sausage batters and in other meat products [40,41,42,43,44,45,46,47,48,49,56]. There was a two-way (*p* < 0.001) interaction between the amount of chitosan and chitosan coating for pH values (Supplementary Material, Table S9). Data for pH related to packaging and storage time are shown in Table S11 (Supplementary Material).

Instrumental mean surface L* (*p* < 0.05), a* (*p* < 0.05), b* (*p* < 0.05), C* (*p* < 0.05), *h* (*p* < 0.05) and λ (*p* < 0.05) values in all 27 tea sausage groups varied between 27.91 (Ch0/UCo/MAP/90) and 32.49 (Ch0/Co/V/45), 9.39 (Ch0/Co/V/90) and 16.48 (Ch0/UCo/MAP/45), 8.28 (Ch0.50/Co/V/45) and 11.84 (Ch0/UCo/MAP/45), 13.23 (Ch0.50/Co/MAP/90) and 20.30 (Ch0/UCo/MAP/45), 33.93 (Ch0.50/0) and 46.60 (Ch0/Co/MAP/45), and 590.9 (Ch0/Co/MAP/45) and 599.0 (Ch0.50/0) nm, respectively (Supplementary Material, Figures S2–S7). The amount of chitosan had no significant effect (*p* > 0.05) on the surface L* values, while chitosan coating, packaging and storage time each had a significant effect (*p* < 0.05) on the surface L* values. Chitosan coating had the effect of increasing surface L* values. The amount of chitosan had no significant effect (*p* > 0.05) on the surface a* values, while chitosan coating, packaging and storage time each had a significant (*p* < 0.05) effect on the surface a* values. Surface a* values on uncoated sausages were higher than on chitosan-coated sausages. The amount of chitosan, chitosan coating, packaging and storage time had significant (*p* < 0.05) effects on the surface b*, C*, *h* and λ values. Tea sausages produced with 0% chitosan had the highest surface b* values, while tea sausages produced with 0.50% chitosan had the lowest surface b* values. Chitosan coating had the effect of decreasing surface b* values. Tea sausages produced with 0 and 0.25% chitosan had higher surface C* values than tea sausages produced with 0.50% chitosan. Also, chitosan coating had the effect of decreasing surface C* values. Tea sausages produced without chitosan had higher surface *h* and lower surface λ values than tea sausages produced with chitosan (0.25 and 0.50%). Chitosan coating had the effect of increasing surface *h* and decreasing λ values (Table 3). There was a two-way (*p* = 0.002–<0.001) interaction between the amount of chitosan and chitosan coating for surface a*, *h* and λ values (Supplementary Material, Table S9). Data for surface colour related to packaging and storage time are shown in Table S11 (Supplementary Material). Further, mean cut cross-section L* (*p* < 0.05), a* (*p* < 0.05), b* (*p* < 0.05), C* (*p* < 0.05), *h* (*p* < 0.05) and λ (*p* < 0.05) values in all 27 tea sausage groups varied between 37.19 (Ch0.50/Co/V/90) and 45.34 (Ch0/0), 9.05 (Ch0.50/UCo/V/90) and 15.49 (Ch0/0), 6.01 (Ch0/UCo/V/90) and 7.61 (Ch0.25/Co/MAP/45), 12.14 (Ch0.50/UCo/V/90) and 17.19 (Ch0/0), 24.07 (Ch0/UCo/V/45) and 41.61 (Ch0.50/UCo/V/90), and 594.9 (Ch0.50/UCo/V/90) and 607.6 (Ch0/UCo/V/45) nm, respectively (Supplementary Material, Figures S8–S13). These results for L*, a* and b* are consistent with those reported in our previous studies [7,8,9]. The amount of chitosan had no significant effect (*p* > 0.05) on the cut cross-section L* values, while chitosan coating, packaging and storage time had significant effects (*p* < 0.05) on the cut cross-section L* values. Chitosan coating had the effect of decreasing cut cross-section L* values. Chitosan coating had no significant effect (*p* > 0.05) on the cut cross-section a* values, while the amount of chitosan, packaging and storage time had significant effects (*p* < 0.05) on the cut cross-section a* values. Tea sausages produced with 0 and 0.25% chitosan had higher cut cross-section a* values than tea sausages produced with 0.50% chitosan. The amount of chitosan and chitosan coating had no significant effect (*p* > 0.05) on the cut cross-section b* and C* values, while packaging and storage time each had a significant effect (*p* < 0.05) on the cut cross-section b* and C* values. Chitosan coating and packaging had no significant effect (*p* > 0.05) on the cut cross-section *h* and λ values, while the amount of chitosan and storage time each had a significant effect (*p* < 0.05) on the cut cross-section h and λ values. Tea sausages produced with 0 and 0.25% chitosan had lower cut cross-section *h* and higher cut cross-section λ values than tea sausages produced with 0.50% chitosan (Table 4). Although statistical differences existed for colour characteristics, sometimes numerical differences were not of great magnitude. There was a two-way (*p* = 0.038–<0.001) interaction between the amount of chitosan and chitosan coating for cut cross-section L*, a*, C*, *h* and λ values (Supplementary Material, Table S9). Data for cut cross-section colour related to packaging and storage time are shown in Table S12 (Supplementary Material). Several previous studies indicate that coating with chitosan and the addition of different amounts of chitosan may affect the colour of the meat products ([14,16,42,43,44,45,47,48,49,50,56,57,58,60], chitosan-coated dry-fermented sausages—*Petrovská klobása* [70], cooked pork sausages with chitosan [71], pork patties with chitosan). However, the colour results of these studies are conflicting or contradictory, which could be explained by various chitosan applications on different meat products. Studies conducted on dried meat products processed with chitosan have shown that chitosan coating produced darker and more red and yellow chitosan-coated dry-fermented sausages—*Petrovská klobása* [14] and chitosan-coated dry-cured meat products—pastirma [59]. On the other hand, Badawy et al. [40,52] and Chang et al. [64] reported that the application of chitosan did not affect the instrumental colour values of meat products.

Mean hardness (*p* < 0.05), springiness (*p* < 0.05), cohesiveness (*p* < 0.05) and chewiness (*p* < 0.05) values in all 27 tea sausage groups varied between 9653.3 (Ch0.50/0) and 20,817.1 g (Ch0.25/Co/MAP/45), 0.389 (Ch0.50/UCo/MAP/45) and 0.514 (Ch0/Co/V/90), 0.402 (Ch0.50/UCo/MAP/90) and 0.513 (Ch0/Co/V/45), and 1854.0 (Ch0.50/0) and 4505.5 (Ch0.25/Co/MAP/45) g, respectively (Supplementary Material, Figures S14–S17). These results are consistent with those reported in our previous studies [7,8,9]. The amount of chitosan, chitosan coating, packaging and storage time had significant (*p* < 0.05) effects on the hardness, springiness, cohesiveness and chewiness values, except chitosan coating on the chewiness. Tea sausages produced with 0 and 0.25% chitosan had higher hardness values than tea sausages produced with 0.50% chitosan. Also, the addition of chitosan decreased the springiness, cohesiveness and chewiness values. Chitosan coating had the effect of decreasing hardness values and increasing springiness and cohesiveness values (Table 5). Although statistical differences existed for springiness and cohesiveness, numerical differences were not of great magnitude. There was a two-way (*p* = 0.030) interaction between the amount of chitosan and chitosan coating for hardness values (Supplementary Material, Table S9). Data for texture related to packaging and storage time are shown in Table S13 (Supplementary Material). Several previous studies indicate that coating with chitosan and the addition of different amounts of chitosan may affect the texture of meat products ([44,45,47,49,50,57,58,70,71,72], meat-based food matrix with chitosan). However, as for colour, the texture results of these studies are conflicting or contradictory, which also could be explained by various chitosan applications on different meat products. On the other hand, Abdallah et al. [60], García et al. [53], Jo et al. [48], Jokanovic et al. [73], chitosan-coated dry-fermented sausage—*Petrovská klobása* and Tirado-Gallegos et al. [56] reported that the application of chitosan did not affect the instrumental texture values of meat products.

### 3.4. Sensory Characteristic of Tea Sausages

Mean overall sensory quality (*p* < 0.05) in all 27 tea sausage groups varied between 4.59 (Ch0/Co/MAP/45) and 4.97 (Ch0/UCo/V/90) (Supplementary Material, Figure S18). The amount of chitosan, chitosan coating, packaging and storage time had significant (*p* < 0.05) effects on the overall sensory quality values. Tea sausages produced with chitosan (0.25 and 0.50%) had higher overall sensory scores than tea sausages produced without chitosan. Chitosan coating had the effect of decreasing overall sensory scores (Table 6). There was a two-way (*p* = 0.004) interaction between the amount of chitosan and chitosan coating for overall sensory quality values (Supplementary Material, Table S9). Data for overall sensory quality related to packaging and storage time are shown in Table S14 (Supplementary Material). It should be noted that statistical significance does not necessarily imply practical or consumer relevance. In the present study, some statistically significant differences in sensory attributes were relatively small in numerical terms. Therefore, these differences should be interpreted with caution and should not automatically be considered indicative of perceptible or meaningful changes in consumer acceptance. Greater emphasis should be placed on differences that were both statistically significant and sufficiently pronounced to have potential practical relevance. Several previous studies indicate that coating with chitosan and the addition of different amounts of chitosan may affect the sensory quality of meat products [15,16,43,45,47,50,51,55,57,59,60,70]. Abdallah et al. [60] observed that the scores received for sensory attributes (appearance, flavour, juiciness, tenderness and overall acceptability) of chitosan-coated pastirma (a dry-cured meat product) were higher than those for traditionally coated. Likewise, Hromiš et al. [15] and Krkić et al. [16] reported that chitosan coating of dry-fermented sausage—*Petrovská klobása* resulted in improved sensory quality (taste and smell or aroma). On the other hand, Jo et al. [48], Carvalho et al. [49], García et al. [53,54], Casquete et al. [63] and Kanatt et al. [67] reported that the application of chitosan did not affect the sensory quality of meat products. In contrast, Arslan and Soyer [51] investigated the effect of chitosan coating on the sensory quality (colour, odour, taste, texture and overall acceptability) of dry-fermented sausage. According to their results, chitosan coating of dry-fermented sausage led to lower taste scores compared to control samples, which is in agreement with our results. In addition, Gökmen and Gürbüz [66] reported that the addition of chitosan to dry-fermented sausages—Turkish sausage (sucuk) did not affect the sensory quality (appearance, colour and flavour), except texture; the addition of much larger amounts of chitosan (1%) affected the texture in a negative way.

### 3.5. Microbiological Characteristics of Tea Sausages

Mean TPC (*p* < 0.05) and LAB (*p* < 0.05) values in all 27 tea sausage groups varied between 3.39 (Ch0/Co/V/90) and 4.76 (Ch0/Co/V/45) and 5.70 (Ch0/Co/V/90) and 8.15 (Ch0.25/0), respectively (Supplementary Material, Figures S19–S20). These results are consistent with those reported in our previous studies [7,8,9]. High LAB counts found in sausages could be explained by the addition of a starter culture to the batters. Chitosan coating and packaging had no significant effects (*p* > 0.05) on the TPC values, while the amount of chitosan and storage time had significant effects (*p* < 0.05) on the TPC values. In addition, the amount of chitosan had no significant effect (*p* > 0.05) on the LAB values, while chitosan coating, packaging and storage time had significant effects (*p* < 0.05) on the LAB values. Tea sausages produced with 0 and 0.25% chitosan had higher TPC values than tea sausages produced with 0.50% chitosan (Table 7), which was consistent with TPC values obtained in sausage batters and with effect of chitosan on the microbiological quality obtained in other meat products [40,41,43,45,46,52,54,55,57,58,59,61,63,64,66,68]. Chitosan coating had the effect of decreasing LAB values (Table 7), which was also consistent with LAB values obtained in sausage batters and with the effect of chitosan on the microbiological quality obtained in other chitosan-coated meat products [47,60,62,65,67]. It is important to emphasize that the reduction in LAB counts observed in the coated sausages should be interpreted with caution. Lactic acid bacteria play an essential role in the fermentation of dry-fermented sausages, contributing to acidification, flavour development, and overall product stability. Therefore, a marked inhibition of LAB growth could potentially be undesirable, particularly during the early stages of fermentation. However, the reduction observed in the present study should not necessarily be interpreted as detrimental, since LAB were still detected in the coated sausages and the coating may have contributed to controlling microbial growth during storage. Thus, the effect of chitosan coating on LAB may be considered concentration- and process-dependent, and its practical significance should be evaluated together with other quality and microbiological parameters. There was a two-way (*p* = 0.001–<0.001) interaction between the amount of chitosan and chitosan coating for TPC and LAB values (Supplementary Material, Table S9). Data for microbiological quality related to packaging and storage time are shown in Table S15 (Supplementary Material).

### 3.6. Principal Component Analysis

**Figure 1:**
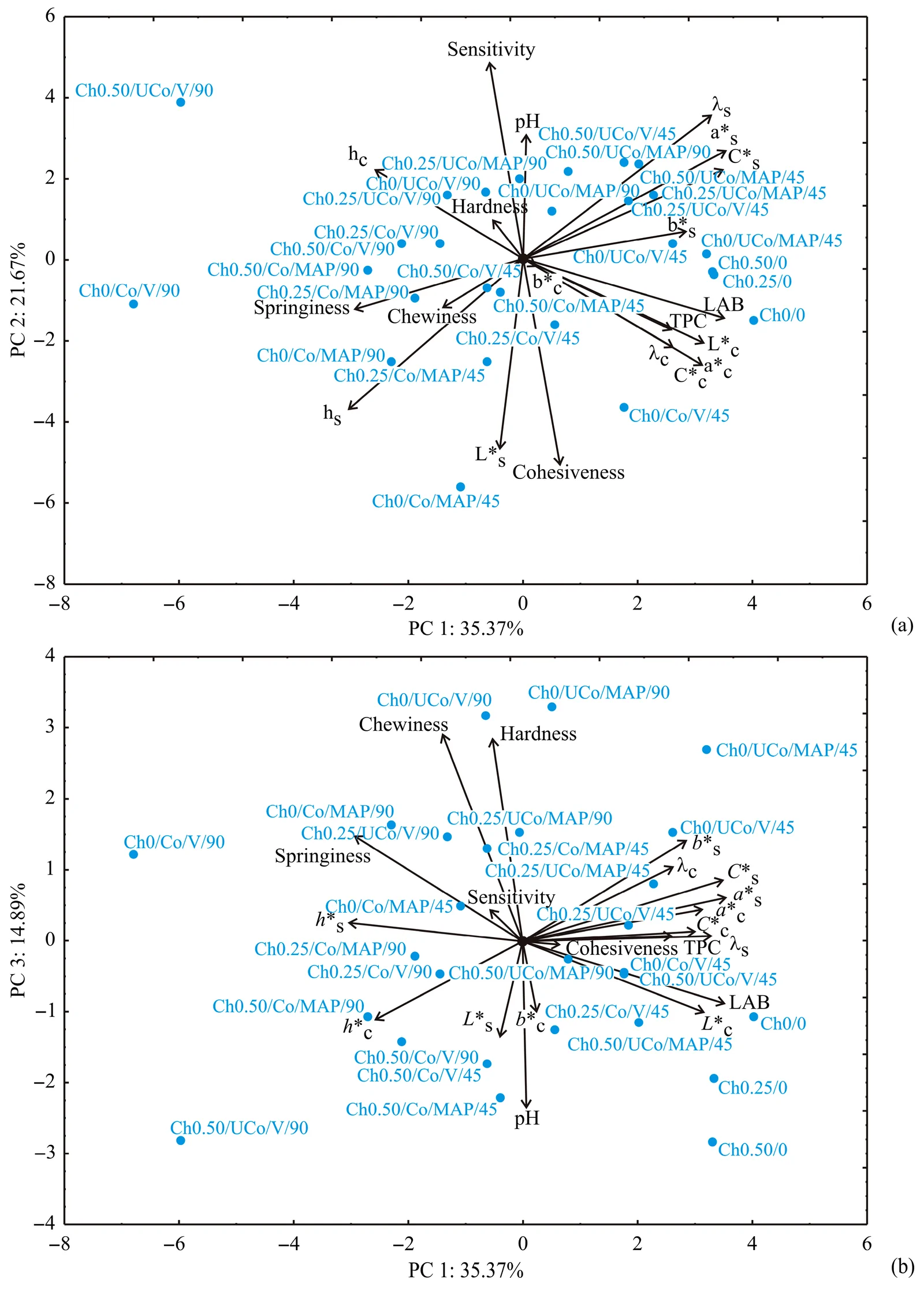
PCA biplot diagram depicting the relationships among physico-chemical, sensory and microbiological parameters: (**a**) projection in PC1–PC2 plane, (**b**) projection in PC1–PC3 plane.

Principal component analysis (PCA) was utilized to explore the relationships among the different samples, as shown in Figure 1. Points located close to each other on the PCA plot indicate similar patterns [74,75]. The first three principal components (PCs) accounted for a substantial portion of the total variance in the dataset, explaining 71.93% collectively. Specifically, the first principal component (PC1) explained 35.37%, the second principal component (PC2) accounted for 21.67%, and the third principal component (PC3) contributed 14.89%, as illustrated in Figure 1. Analysis of the variable projections on the factor plane revealed that LAB values had a significant positive influence on the PC1 coordinate, representing 9.77% of the total variance based on correlation, alongside surface colour a* (9.96%), C* (9.64%) and λ (8.61%) values, as well as cut cross-section colour L* (7.98%), a* (7.81%) and C* (7.20%) values. The most substantial negative influence among surface colour parameters on the PC1 coordinate was from *h* (7.32%). The PC2 coordinate was positively influenced by overall sensory quality (15.40%) and surface colour λ (8.23%) values. Negative influences on the PC2 coordinate included cohesiveness (16.46%) and surface colour L* (14.03%) and *h* (8.74%) values. The PC3 coordinate was positively influenced by hardness (23.38%) and chewiness (24.20%). Negative influences on the PC3 coordinate included pH value (15.92%).

### 3.7. Study Limitations and Future Perspectives

The present study has several limitations that should be considered when interpreting the results. First, the physico-chemical and compositional characteristics of the raw meat used for sausage production were not determined. Although the characteristics of the sausage batter are presented in Table 1, the lack of detailed characterization of the raw meat represents a limitation of the study, as the initial quality and composition of the raw material may influence the physico-chemical, microbiological, and sensory properties of the final product.

Second, the present study focused on selected physico-chemical, textural, microbiological, and sensory characteristics of tea sausage. The effects of chitosan and chitosan coating on other important quality parameters, such as oxidative stability, microstructure, and fatty acid composition, were not evaluated. These parameters could provide additional information on the protective effects of chitosan and its potential influence on the nutritional and structural quality of fermented sausages.

Third, the microbiological evaluation was limited to total plate counts (TPC), lactic acid bacteria (LAB), and the selected microbiological groups investigated in the study. Other microbiologically relevant groups and specific spoilage microorganisms were not investigated. Therefore, the present results provide only a partial assessment of the microbiological effects of chitosan and chitosan coating during storage. In particular, the absence of significant changes in some microbiological parameters should be interpreted with caution, as changes in other microbial groups may have occurred but were not assessed. Future studies should therefore include a broader microbiological characterization, including the dynamics of relevant spoilage and/or pathogenic microorganisms, as well as, where appropriate, the identification of individual microbial groups or species using more advanced microbiological or molecular approaches.

Fourth, the sensory evaluation was conducted in a single evaluation session; therefore, the reproducibility of the sensory panel across independent sessions could not be assessed. Although the sensory evaluation provided useful information on the acceptability and sensory characteristics of the sausages, repeated sensory evaluations would provide stronger evidence regarding the consistency and reproducibility of the observed differences. In addition, some statistically significant differences between treatments were relatively small in numerical terms and may therefore have limited practical relevance from a consumer perspective. Future studies should include repeated sensory evaluation sessions and, preferably, a larger and appropriately trained panel to provide more robust information on the reproducibility and practical significance of the observed sensory differences.

Future studies should therefore include a more detailed characterization of the raw meat and sausage batter, as well as an assessment of oxidative stability, microstructural changes, fatty acid profiles, and a broader range of microbiological parameters during storage. Further research should also investigate the interactions between chitosan, meat components, and different packaging conditions. Evaluating a broader range of chitosan concentrations and coating formulations, together with different storage conditions and longer storage periods, could provide further insight into the potential of chitosan as a natural strategy for maintaining the quality of fermented sausages. In addition, more comprehensive microbiological analyses would help clarify the mechanisms underlying the antimicrobial effects of chitosan and determine whether its application selectively affects specific microbial groups during fermentation and storage. Such studies would contribute to a more comprehensive understanding of the technological, nutritional, microbiological, sensory, and functional effects of chitosan and help determine its practical applicability in the production and preservation of fermented meat products.

## 4. Conclusions

By investigating the effects of adding different amounts of chitosan powder (0.25 and 0.50%) to the batter and applying chitosan coating of tea sausage (dry-fermented sausage) on the physico-chemical (pH, surface and cut cross-section colour: L*, a*, b*, C*, *h* and λ, hardness, springiness, cohesiveness and chewiness), overall sensory and microbiological (TPC and LAB) quality during 90 days of storage under vacuum and MAP conditions, the following were concluded. The adding of chitosan increased the pH, surface dominant wavelength (λ) and hue angle (*h*, 0.50%) of the cut cross-section and the overall sensory quality, and decreased the surface yellowness (b*), chroma-saturation index (C*, 0.50%) and hue angle (*h*), and redness (a*, 0.50%) and dominant wavelength (λ, 0.50%) of the cut cross-section, as well as the hardness (0.50%), springiness, cohesiveness, chewiness and TPC (0.50%). The chitosan coating increased surface lightness (L*) and hue angle (*h*) and springiness and cohesiveness, and decreased the surface redness (a*), yellowness (b*), chroma-saturation index (C*) and dominant wavelength (λ), and lightness (L*) of the cut cross-section, as well as the hardness, overall sensory quality and LAB.

In addition, the packaging (vacuum and MAP) increased the surface hue angle (*h*), hardness, springiness and chewiness and the overall sensory quality (vacuum), and decreased the surface lightness (L*, MAP), redness (a*), yellowness (b*), chroma-saturation index (C*) and dominant wavelength (λ), and the lightness (L*), redness (a*), yellowness (b*, vacuum) and chroma-saturation index (C*) of the cut cross-section, as well as the cohesiveness and LAB compared to unpacked sausage. The MAP packaging increased the lightness (L*), redness (a*), yellowness (b*) and chroma-saturation index (C*) of the cut cross-section and the hardness, and decreased the pH value.

During storage, the surface hue angle (*h*), hardness and chewiness increased up to 45 days, the hue angle (*h*) of the cut cross-section, springiness and overall sensory quality increased from day 45 to day 90, the dominant wavelength (λ) of the cut cross-section increased up to 45 days and then decreased up to 90 days, the yellowness (b*) of the cut cross-section decreased up to 45 days, the surface lightness (L*) and TPC decreased from day 45 to day 90, while the surface redness (a*), yellowness (b*), chroma-saturation index (C*) and dominant wavelength (λ), and lightness (L*), redness (a*) and chroma-saturation index (C*) of the cut cross-section, cohesiveness and LAB decreased steadily to day 90.

Overall, the results indicate that chitosan addition and chitosan coating exerted different effects on the quality characteristics of tea sausage. Chitosan addition, particularly at the higher concentration, showed more consistent positive effects on overall sensory quality and microbial stability, whereas chitosan coating had a more pronounced influence on colour characteristics and LAB counts. The effects of both chitosan application strategies were also dependent on the packaging conditions and storage time. These findings indicate that the mode of chitosan application is an important factor determining its technological effects and should be considered when developing chitosan-based strategies for improving the quality and stability of dry-fermented sausages.

## Figures and Tables

**Table 1:** Characterization [pH value, chemical (aggregate samples) and microbiological characteristics (aggregate samples)] of tea sausage batters.

Traits ^1^	Ch0/B	Ch0.25/B	Ch0.50/B
pH	6.01 ± 0.01 ^c^	6.26 ± 0.01 ^b^	6.45 ± 0.01 ^a^
Moisture (g/100 g)	57.70	58.60	58.44
Protein (g/100 g)	14.00	14.13	14.28
Total fat (g/100 g)	23.03	21.82	21.73
Total ash (g/100 g)	3.28	3.31	3.27
Chloride (g/100 g)	2.51	2.56	2.53
Total phosphorus (g/kg)	3.91	3.98	3.78
Nitrite (mg/kg)	72.5	70.3	77.0
TPC (log CFU/g)	4.30 ± 1.45 ^a^	3.99 ± 1.56 ^c^	4.16 ± 1.51 ^b^
LAB (log CFU/g)	5.99 ± 2.02 ^a^	5.04 ± 1.78 ^c^	5.40 ± 1.77 ^b^

^1^ Results for pH, TPC and LAB are reported as means ± standard errors of the mean. The chemical composition parameters of the batters were determined based on a single measurement, without repetition. TPC—total plate count; LAB—lactic acid bacteria; CFU—colony-forming unit; ^abc^ *p* < 0.05.

**Table 2:** Characterization (chemical characteristics of aggregate samples) of tea sausages.

Traits ^1^	Ch0/0	Ch0.25/0	Ch0.50/0
Moisture (g/100 g)	33.70	33.54	33.62
Protein (g/100 g)	22.40	22.74	22.14
Total fat (g/100 g)	36.84	36.22	36.73
Total ash (g/100 g)	5.25	5.33	5.40
Chloride (g/100 g)	4.02	4.12	4.08
Total phosphorus (g/kg)	6.26	5.97	6.35
Nitrite (mg/kg)	4.8	4.0	4.3

^1^ The chemical composition parameters of the tea sausages were determined based on a single measurement, without repetition.

**Table 3:** pH value and surface colour characteristics of tea sausage.

Treatments ^1^	pH	L*	a*	b*	C*	*h*	λ (nm)
Effect of amount of chitosan (AC)							
0%	5.36 ± 0.02 ^c^	30.2 ± 0.23	13.4 ± 0.31	10.6 ± 0.12 ^a^	17.1 ± 0.29 ^a^	38.9 ± 0.57 ^a^	595.7 ± 0.36 ^b^
0.25%	5.47 ± 0.01 ^b^	30.1 ± 0.20	13.5 ± 0.24	9.9 ± 0.14 ^b^	16.8 ± 0.27 ^a^	36.4 ± 0.27 ^b^	597.0 ± 0.21 ^a^
0.50%	5.57 ± 0.01 ^a^	30.0 ± 0.18	12.8 ± 0.28	9.4 ± 0.15 ^c^	16.0 ± 0.30 ^b^	36.7 ± 0.36 ^b^	596.7 ± 0.26 ^a^
*p* value	<0.001	0.638	0.177	<0.001	0.013	<0.001	0.005
Effect of chitosan coating (CC)							
Uncoated	5.48 ± 0.02	29.4 ± 0.15 ^b^	15.0 ± 0.14 ^a^	10.6 ± 0.10 ^a^	18.4 ± 0.16 ^a^	35.3 ± 0.15 ^b^	598.1 ± 0.11 ^a^
Coated	5.45 ± 0.02	31.0 ± 0.15 ^a^	11.1 ± 0.18 ^b^	9.2 ± 0.10 ^b^	14.5 ± 0.18 ^b^	39.9 ± 0.43 ^a^	594.4 ± 0.24 ^b^
*p* value	0.176	<0.001	<0.001	<0.001	<0.001	<0.001	<0.001

^1^ Results are reported as means ± standard errors of the mean. L*—a measure of darkness/lightness (higher value indicates a lighter colour); a*—a measure of redness (higher value indicates a redder colour); b*—a measure of yellowness (higher value indicates a more yellow colour); *h*—hue angle (lower values indicates a redder colour); C*—saturation index (higher values indicates greater saturation of red); λ—dominant wavelength (orange colour: 585–620 nm); ^abc^ *p* < 0.05.

**Table 4:** Cut cross-section colour characteristics of tea sausage.

Treatments ^1^	L*	a*	b*	C*	*h*	λ (nm)
Effect of amount of chitosan (AC)						
0%	41.0 ± 0.27	14.0 ± 0.14 ^a^	6.8 ± 0.08	15.6 ± 0.15	25.9 ± 0.25 ^b^	605.4 ± 0.29 ^a^
0.25%	41.6 ± 0.26	13.9 ± 0.11 ^a^	6.9 ± 0.08	15.6 ± 0.13	26.3 ± 0.17 ^b^	604.6 ± 0.21 ^a^
0.50%	40.7 ± 0.27	13.4 ± 0.19 ^b^	7.0 ± 0.09	15.2 ± 0.17	28.1 ± 0.61 ^a^	603.5 ± 0.40 ^b^
*p* value	0.051	0.012	0.127	0.165	<0.001	<0.001
Effect of chitosan coating (CC)						
Uncoated	41.5 ± 0.21 ^a^	13.8 ± 0.13	6.9 ± 0.06	15.5 ± 0.12	27.0 ± 0.37	604.4 ± 0.26
Coated	40.5 ± 0.22 ^b^	13.8 ± 0.12	6.9 ± 0.07	15.4 ± 0.13	26.5 ± 0.21	604.6 ± 0.24
*p* value	0.001	0.991	0.501	0.708	0.301	0.571

^1^ Results are reported as means ± standard errors of the mean. L*—a measure of darkness/lightness (higher value indicates a lighter colour); a*—a measure of redness (higher value indicates a redder colour); b*—a measure of yellowness (higher value indicates a more yellow colour); *h*—hue angle (lower values indicates a redder colour); C*—saturation index (higher values indicates greater saturation of red); λ—dominant wavelength (orange colour: 585–620 nm); ^ab^ *p* < 0.05.

**Table 5:** Texture characteristics of tea sausage.

Treatments ^1^	Hardness (g)	Springiness	Cohesiveness	Chewiness (g)
Effect of amount of chitosan (AC)				
0%	16,761.6 ± 292.5 ^a^	0.459 ± 0.004 ^a^	0.467 ± 0.003 ^a^	3571.2 ± 61.9 ^a^
0.25%	16,109.6 ± 337.9 ^a^	0.443 ± 0.004 ^b^	0.447 ± 0.003 ^b^	3181.3 ± 72.0 ^b^
0.50%	14,003.0 ± 265.1 ^b^	0.428 ± 0.004 ^c^	0.430 ± 0.003 ^c^	2551.6 ± 44.0 ^c^
*p* value	<0.001	<0.001	<0.001	<0.001
Effect of chitosan coating (CC)				
Uncoated	16,063.0 ± 269.2 ^a^	0.432 ± 0.003 ^b^	0.437 ± 0.003 ^b^	3028.1 ± 56.1
Coated	15,071.3 ± 245.0 ^b^	0.457 ± 0.004 ^a^	0.461 ± 0.003 ^a^	3193.9 ± 66.8
*p* value	0.009	<0.001	<0.001	0.058

^1^ Results are reported as means ± standard errors of the mean. ^abc^ *p* < 0.05.

**Table 6:** Overall sensory quality of tea sausage.

Treatments ^1^	Overall Sensory Quality
Effect of amount of chitosan (AC)	
0%	4.81 ± 0.02 ^b^
0.25%	4.87 ± 0.02 ^a^
0.50%	4.87 ± 0.01 ^a^
*p* value	0.019
Effect of chitosan coating (CC)	
Uncoated	4.88 ± 0.01 ^a^
Coated	4.82 ± 0.02 ^b^
*p* value	0.004

^1^ Results are reported as means ± standard errors of the mean. ^ab^ *p* < 0.05.

**Table 7:** Microbiological characteristics of tea sausage.

Treatments ^1^	TPC (log CFU/g)	LAB (log CFU/g)
Effect of amount of chitosan (AC)		
0%	4.16 ± 0.06 ^a^	7.06 ± 0.09
0.25%	4.13 ± 0.02 ^a^	6.96 ± 0.10
0.50%	4.00 ± 0.05 ^b^	7.05 ± 0.08
*p* value	0.022	0.679
Effect of chitosan coating (CC)		
Uncoated	4.14 ± 0.02	7.15 ± 0.07 ^a^
Coated	4.05 ± 0.02	6.86 ± 0.06 ^b^
*p* value	0.064	0.005

^1^ Results are reported as means ± standard errors of the mean. TPC—total plate count; LAB—lactic acid bacteria; CFU—colony forming units; ^ab^ *p* < 0.05.

## Data Availability

The original contributions presented in this study are included in the article/Supplementary Materials. Further inquiries can be directed to the corresponding author.

## Supplementary Materials

The following supporting information can be downloaded at: https://www.mdpi.com/article/10.3390/foods15173125/s1, Table S1. Conditions during smoking, fermentation, drying and ripening of tea sausages; Table S2. Groups of manufactured tea sausages and their marks (experimental design); Table S3. ANOVA analysis for pH value of tea sausage; Table S4. ANOVA analysis for surface colour characteristics of tea sausage; Table S5. ANOVA analysis for cut cross section colour characteristics of tea sausage; Table S6. ANOVA analysis for texture characteristics of tea sausage; Table S7. ANOVA analysis for overall sensory quality of tea sausage; Table S8. ANOVA analysis for microbiological characteristics of tea sausage; Table S9. The effect of two-way. three-way and four-way interactions among processing parameters on the quality of tea sausages (dry fermented sausages) expressed as *p* value; Table S10. The effect of two-way. three-way and four-way interactions among processing parameters on the quality of tea sausages (dry fermented sausages) expressed as *p* value (continued); Table S11. pH value and surface colour characteristics of tea sausage; Table S12. Cut cross section colour characteristics of tea sausage; Table S13. Texture characteristics of tea sausage; Table S14. Overall sensory quality of tea sausage; Table S15. Microbiological characteristics of tea sausage; Figure S1. The effect of amount of chitosan, chitosan coating, packaging and storage time on pH values (mean ± SE) of tea sausages, ^abcdefghi^ *p* < 0.05; Figure S2. The effect of amount of chitosan, chitosan coating, packaging and storage time on surface *L** values (mean ± SE) of tea sausages, ^abcdefgh^ *p* < 0.05; Figure S3. The effect of amount of chitosan, chitosan coating, packaging and storage time on surface *a** values (mean ± SE) of tea sausages, ^abcdefgh^ *p* < 0.05; Figure S4. The effect of amount of chitosan, chitosan coating, packaging and storage time on surface *b** values (mean ± SE) of tea sausages, ^abcdefghi^ *p* < 0.05; Figure S5. The effect of amount of chitosan, chitosan coating, packaging and storage time on surface *C** values (mean ± SE) of tea sausages, ^abcdefghi^ *p* < 0.05; Figure S6. The effect of amount of chitosan, chitosan coating, packaging and storage time on surface *h* values (mean ± SE) of tea sausages, ^abcdef^ *p* < 0.05; Figure S7. The effect of amount of chitosan, chitosan coating, packaging and storage time on surface λ values (mean ± SE) of tea sausages, ^abcdefghij^ *p* < 0.05; Figure S8. The effect of amount of chitosan, chitosan coating, packaging and storage time on cut cross section *L** values (mean ± SE) of tea sausages, ^abcdefghij^ *p* < 0.05; Figure S9. The effect of amount of chitosan, chitosan coating, packaging and storage time on cut cross section *a** values (mean ± SE) of tea sausages, ^abcd^ *p* < 0.05; Figure S10. The effect of amount of chitosan, chitosan coating, packaging and storage time on cut cross section *b** values (mean ± SE) of tea sausages, ^abcdef^ *p* < 0.05; Figure S11. The effect of amount of chitosan, chitosan coating, packaging and storage time on cut cross section *C** values (mean ± SE) of tea sausages, ^abcde^ *p* < 0.05; Figure S12. The effect of amount of chitosan, chitosan coating, packaging and storage time on cut cross section *h* values (mean ± SE) of tea sausages, ^abc^ *p* < 0.05; Figure S13. The effect of amount of chitosan, chitosan coating, packaging and storage time on cut cross section λ values (mean ± SE) of tea sausages, ^abcdef^ *p* < 0.05; Figure S14. The effect of amount of chitosan, chitosan coating, packaging and storage time on hardness (g) values (mean ± SE) of tea sausages, ^abcdefghijklmno^ *p* < 0.05; Figure S15. The effect of amount of chitosan, chitosan coating, packaging and storage time on springiness values (mean ± SE) of tea sausages, ^abcdefghij^ *p* < 0.05; Figure S16. The effect of amount of chitosan, chitosan coating, packaging and storage time on cohesiveness values (mean ± SE) of tea sausages, ^abcdefghijkl^ *p* < 0.05; Figure S17. The effect of amount of chitosan, chitosan coating, packaging and storage time on chewiness (g) values (mean ± SE) of tea sausages, ^abcdefghijk^ *p* < 0.05; Figure S18. The effect of amount of chitosan, chitosan coating, packaging and storage time on overall sensory quality values (mean ± SE) of tea sausages, ^ab^ *p* < 0.05; Figure S19. The effect of amount of chitosan, chitosan coating, packaging and storage time on TPC (log cfu/g) values (mean ± SE) of tea sausages, TPC—total plate count; CFU—colony forming units, ^abcdefghijk^ *p* < 0.05; Figure S20. The effect of amount of chitosan, chitosan coating, packaging and storage time on LAB (log cfu/g) values (mean ± SE) of tea sausages, LAB—lactic acid bacteria; CFU—colony forming units, ^abcdefghijklmno^ *p* < 0.05.
