# Supplementary material for: The Effect of Addition of Different Amounts of Chitosan Powder to the Batter and Chitosan Coating of Tea Sausage (Dry-Fermented Sausage) on the Physico-Chemical, Sensory and Microbiological Quality
Source: Foods. 2026 Sep 2;15(17):3125. doi: 10.3390/foods15173125 (PMC13565233; doi:10.3390/foods15173125)
Supplement: Supplementary file 1 [file foods-15-03125-s001.zip › foods-4361539-supplementary.pdf]

Supplementary material

The Effect of Addition of Different Amounts of Chitosan Powder to the Batter and Chitosan Coating of Tea Sausage (Dry Fermented Sausage) on the Physico-Chemical, Sensory and Microbiological Quality

Jelena Latinović [1,2], Vladimir Tomović [1,*], Lato Pezo [3], Senka Popović [1], Nevena Hromiš [1], Sunčica Kocić-Tanackov [1], Aleksandra Martinović [4], Dragan Vujadinović [5] and Vesna Đorđević [6]

[1] Faculty of Technology Novi Sad, University of Novi Sad, 21000 Novi Sad, Serbia; jelenarlatinovic@gmail.com (J.L.); madjarev@uns.ac.rs (S.P.); nevena.krkic@uns.ac.rs (N.H.); suncicat@uns.ac.rs (S.K.-T.)
[2] Ribella Foods Doo, 21234 Bački Jarak, Serbia
[3] Institute of General and Physical Chemistry, University of Belgrade, 11158 Belgrade, Serbia; latopezo@yahoo.co.uk
[4] Faculty for Food Technology, Food Safety and Ecology, University of Donja Gorica, Donja Gorica, 81000 Podgorica, Montenegro; aleksandra.martinovic@udg.edu.me
[5] Faculty of Technology Zvornik, University of East Sarajevo, 75400 Zvornik, Bosnia and Herzegovina; dragan.vujadinovic@tfzv.ues.rs.ba
[6] Institute of Meat Hygiene and Technology, 11040 Belgrade, Serbia; vesna.djordjevic@inmes.rs
* Correspondence: tomovic@uns.ac.rs

Table S1. Conditions during smoking, fermentation, drying and ripening of tea sausages.

Day	T_{db} (°C)	Relative humidity (%)	T_{wb} (°C)	Smoking (h)	Air velocity (Hz)
Overnight	20	90	18.9		40 - 50
Smoking and fermentation					
1	18	88	16.7	3 x 3	40 - 50
2	18	86	16.5	3 x 3	40 - 50
3	18	84	16.3	3 x 3	40 - 50
4	18	82	16.0	3 x 3	40 - 50
5	16	80	13.9	3 x 3	40 - 50
Drying and ripening					
6	15 - 16	80 - 78	12.8 - 13.7		35 - 47
7	15 - 16	78 - 76	12.8 - 12.6		35 - 47
8	15 - 16	76 - 74	12.6 - 12.4		35 - 47
9	15 - 16	74	12.4		35 - 47
10	15 - 16	74	12.4		35 - 47
11	15 - 16	74	12.4		35 - 47
12	15 - 16	74	12.4		35 - 47
13	15 - 16	74	12.4		35 - 47
14	15 - 16	74 - 72	12.4 - 12.2		35 - 47
15	15 - 16	74 - 72	12.4 - 12.2		35 - 47
16	15 - 16	74 - 72	12.4 - 12.2		35 - 47
17	15 - 16	72 - 70	12.2 - 12.0		35 - 47
18	15 - 16	70 - 72	12.0 - 12.2		35 - 47
19	15 - 16	70	12.0		35 - 47
20	15 - 16	70	12.0		35 - 47
21	15 - 16	70	12.0		35 - 47

T_{db} – dry-bulb temperature; T_{wb} – wet-bulb temperature.

Table S2. Groups of manufactured tea sausages and their marks (experimental design).

Mark	Description
Ch0/B	Amounts of chitosan in sausage stuffing: 0%; Day of sampling: raw meat/fat mixture (batter)
Ch0.25/B	Amounts of chitosan in sausage stuffing: 0.25%; Day of sampling: raw meat/fat mixture (batter)
Ch0.50/B	Amounts of chitosan in sausage stuffing: 0.50%; Day of sampling: raw meat/fat mixture (batter)
Ch0/0	Amounts of chitosan in sausage stuffing: 0%; Day of sampling: 26 days after stuffing (at the end of drying process)
Ch0.25/0	Amounts of chitosan in sausage stuffing: 0.25%; Day of sampling: 26 days after stuffing (at the end of drying process)
Ch0.50/0	Amounts of chitosan in sausage stuffing: 0.50%; Day of sampling: 26 days after stuffing (at the end of drying process)
Ch0/UCo/V/45	Amounts of chitosan in sausage stuffing: 0%; Uncoated: UCo; Packaging method: V; Day of sampling: after 45 days of storage
Ch0/UCo/MAP/45	Amounts of chitosan in sausage stuffing: 0%; Uncoated: UCo; Packaging method: MAP; Day of sampling: after 45 days of storage
Ch0/Co/V/45	Amounts of chitosan in sausage stuffing: 0%; Coated: Co; Packaging method: V; Day of sampling: after 45 days of storage
Ch0/Co/MAP/45	Amounts of chitosan in sausage stuffing: 0%; Coated: Co; Packaging method: MAP; Day of sampling: after 45 days of storage
Ch0.25/UCo/V/45	Amounts of chitosan in sausage stuffing: 0.25%; Uncoated: UCo; Packaging method: V; Day of sampling: after 45 days of storage
Ch0.25/UCo/MAP/45	Amounts of chitosan in sausage stuffing: 0.25%; Uncoated: UCo; Packaging method: MAP; Day of sampling: after 45 days of storage
Ch0.25/Co/V/45	Amounts of chitosan in sausage stuffing: 0.25%; Coated: Co; Packaging method: V; Day of sampling: after 45 days of storage
Ch0.25/Co/MAP/45	Amounts of chitosan in sausage stuffing: 0.25%; Coated: Co; Packaging method: MAP; Day of sampling: after 45 days of storage
Ch0.50/UCo/V/45	Amounts of chitosan in sausage stuffing: 0.50%; Uncoated: UCo; Packaging method: V; Day of sampling: after 45 days of storage
Ch0.50/UCo/MAP/45	Amounts of chitosan in sausage stuffing: 0.50%; Uncoated: UCo; Packaging method: MAP; Day of sampling: after 45 days of storage
Ch0.50/Co/V/45	Amounts of chitosan in sausage stuffing: 0.50%; Coated: Co; Packaging method: V; Day of sampling: after 45 days of storage
Ch0.50/Co/MAP/45	Amounts of chitosan in sausage stuffing: 0.50%; Coated: Co; Packaging method: MAP; Day of sampling: after 45 days of storage
Ch0/UCo/V/90	Amounts of chitosan in sausage stuffing: 0%; Uncoated: UCo; Packaging method: V; Day of sampling: after 90 days of storage
Ch0/UCo/MAP/90	Amounts of chitosan in sausage stuffing: 0%; Uncoated: UCo; Packaging method: MAP; Day of sampling: after 90 days of storage
Ch0/Co/V/90	Amounts of chitosan in sausage stuffing: 0%; Coated: Co; Packaging method: V; Day of sampling: after 90 days of storage
Ch0/Co/MAP/90	Amounts of chitosan in sausage stuffing: 0%; Coated: Co; Packaging method: MAP; Day of sampling: after 90 days of storage
Ch0.25/UCo/V/90	Amounts of chitosan in sausage stuffing: 0.25%; Uncoated: UCo; Packaging method: V; Day of sampling: after 90 days of storage
Ch0.25/UCo/MAP/90	Amounts of chitosan in sausage stuffing: 0.25%; Uncoated: UCo; Packaging method: MAP; Day of sampling: after 90 days of storage
Ch0.25/Co/V/90	Amounts of chitosan in sausage stuffing: 0.25%; Coated: Co; Packaging method: V; Day of sampling: after 90 days of storage
Ch0.25/Co/MAP/90	Amounts of chitosan in sausage stuffing: 0.25%; Coated: Co; Packaging method: MAP; Day of sampling: after 90 days of storage
Ch0.50/UCo/V/90	Amounts of chitosan in sausage stuffing: 0.50%; Uncoated: UCo; Packaging method: V; Day of sampling: after 90 days of storage
Ch0.50/UCo/MAP/90	Amounts of chitosan in sausage stuffing: 0.50%; Uncoated: UCo; Packaging method: MAP; Day of sampling: 90
Ch0.50/Co/V/90	Amounts of chitosan in sausage stuffing: 0.50%; Coated: Co; Packaging method: V; Day of sampling: after 90 days of storage
Ch0.50/Co/MAP/90	Amounts of chitosan in sausage stuffing: 0.50%; Coated: Co; Packaging method: MAP; Day of sampling: after 90 days of storage

ANOVA analysis – pH value

The ANOVA results for pH indicate that all main fixed effects – storage time (ST), amount of chitosan (AC), chitosan coating (CC) and packaging (P) – had statistically significant influences ($p < 0.05$), highlighting their individual importance in modulating pH during the study. Among the interactions, a highly significant effect was observed for AC × CC ($p < 0.05$), suggesting a synergistic influence of chitosan concentration and coating presence on pH values. Additionally, the interaction AC × P was also significant ($p < 0.05$), indicating that the impact of chitosan amount on pH depends on the type of packaging applied. Other interaction terms, including ST × AC, ST × CC, ST × P and CC × P, were not statistically significant ($P > 0.05$), suggesting minimal combined effects among these factors. Regarding random effects, most were non-significant ($P > 0.05$), except RP × ST, which was significant ($p < 0.05$), implying some variation in the ST effect across repetitions. Overall, these results emphasize that pH is significantly influenced by individual and combined effects of chitosan application and packaging, with storage time playing a dominant role (Table S3).

Table S3. ANOVA analysis for pH value of tea sausage.

	Effect	pH
Intercept	Fixed	< 0.001
RP	Random	0.362
ST	Fixed	< 0.001
AC	Fixed	< 0.001
CC	Fixed	< 0.001
P	Fixed	< 0.001
RP × ST	Random	0.010
RP × AC	Random	0.985
ST × AC	Fixed	0.117
RP × CC	Random	0.398
ST × CC	Fixed	0.905
AC × CC	Fixed	< 0.001
RP × P	Random	0.957
ST × P	Fixed	0.905
AC × P	Fixed	0.007
CC × P	Fixed	0.087

AC – amount of chitosan; CC – chitosan coating; P – packaging; ST – storage time; RP – repetitions.

ANOVA analysis – Surface colour characteristics of tea sausage

The univariate ANOVA for surface colour parameters (L^*, a^*, b^*, C^*, h and λ) revealed several statistically significant main and interaction effects. Storage time (ST) significantly influenced all colour attributes ($P > 0.05$) except a^*, with P values of < 0.001 for L^*, b^*, C^*, h and λ, indicating a strong temporal effect on surface colour stability. The amount of chitosan (AC) had significant effects on a^* ($p < 0.05$), h ($p < 0.05$) and λ ($p < 0.05$), while the chitosan coating (CC) was highly significant for L^* ($p < 0.05$), a^* ($p < 0.001$), b^* ($p < 0.001$) and C^* ($p < 0.001$), suggesting its pronounced influence on surface redness and chromaticity. Packaging (P) significantly affected a^*, b^* and C^* (all $p < 0.001$), but not L^*, h or λ ($P > 0.05$). Among interaction terms, AC × CC was highly significant for L^* ($p < 0.05$), a^* ($p < 0.001$), C^* ($p < 0.001$), h ($p < 0.05$) and λ ($p < 0.05$), indicating a synergistic effect between the amount and form of chitosan application. The ST × CC interaction also significantly influenced a^*, C^*, h and λ (all $p < 0.001$), confirming that the impact of the coating is storage-time dependent. Furthermore, AC × P affected a^* ($p < 0.01$), C^* ($p < 0.05$), h ($p < 0.05$) and λ ($p < 0.01$), CC × P was significant for a^* ($p < 0.001$), C^* ($p < 0.01$), h ($p < 0.05$) and λ ($p < 0.05$), and ST × P was significant for a^* ($p < 0.01$), C^* ($p < 0.05$) and h ($p < 0.05$), further demonstrating the importance of chitosan–packaging interactions on colour attributes. In contrast, all random effects and their interactions (e.g., RP, RP × ST, RP × AC, RP × CC, RP × P) were non-significant across all parameters ($P > 0.05$), indicating that the variability introduced by repetitions and their interactions was negligible. These results collectively highlight that surface colour is primarily influenced by storage conditions and chitosan-related treatments, both independently and in interaction with packaging (Table S4).

Table S4. ANOVA analysis for surface colour characteristics of tea sausage.

	Effect	L^*	a^*	b^*	C^*	h	λ
Intercept	Fixed	< 0.001	< 0.001	< 0.001	< 0.001	< 0.001	< 0.001
RP	Random	1.000	0.639	0.895	0.212	1.000	1.000
ST	Fixed	< 0.001	0.063	< 0.001	< 0.001	< 0.001	< 0.001
AC	Fixed	0.733	0.043	0.138	0.360	0.015	0.025
CC	Fixed	0.030	< 0.001	< 0.001	< 0.001	0.305	0.103
P	Fixed	0.375	< 0.001	< 0.001	< 0.001	0.329	0.174
RP × ST	Random	1.000	0.364	0.977	0.212	0.998	0.999
RP × AC	Random	1.000	0.997	0.768	0.861	0.995	0.998
ST × AC	Fixed	0.118	0.747	0.077	0.747	0.067	0.120
RP × CC	Random	1.000	0.499	0.991	0.314	1.000	1.000
ST × CC	Fixed	0.701	< 0.001	0.072	< 0.001	< 0.001	< 0.001
AC × CC	Fixed	0.002	< 0.001	0.059	< 0.001	0.009	0.012
RP × P	Random	1.000	0.885	0.858	0.766	0.816	0.959
ST × P	Fixed	0.810	0.001	0.545	0.003	0.049	0.162
AC × P	Fixed	0.680	0.001	0.631	0.021	0.002	0.001
CC × P	Fixed	0.636	< 0.001	0.709	0.001	0.005	0.012

AC – amount of chitosan; CC – chitosan coating; P – packaging; ST – storage time; RP – repetitions.

ANOVA analysis – Cut cross section characteristics of tea sausage

The univariate ANOVA results for the cut cross-section colour parameters (L^*, a^*, b^*, C^*, h and λ) reveal several statistically significant effects. The storage time (ST) showed highly significant effects on all parameters except b^* ($P > 0.05$), with P values for L^*, a^*, C^*, h and λ all equal to $p < 0.001$, indicating that colour attributes, were strongly influenced by storage duration. The amount of chitosan (AC) significantly affected L^* ($p < 0.05$), a^* ($p < 0.05$) and C^* ($p < 0.04$), while the effects on b^*, h and λ were not significant ($p < 0.05$). The chitosan coating (CC) had a significant effect only on a^* ($p < 0.05$) and C^* ($p < 0.01$), with non-significant influence on L^*, b^*, h and λ ($P > 0.05$). In contrast, packaging (P) exhibited a highly significant effect on L^*, a^*, b^* and C^* (all $p < 0.001$), but was not significant for h or λ ($P > 0.05$). Among interaction effects, ST × AC significantly affected a^* ($p < 0.05$), h ($p < 0.01$) and λ ($p < 0.01$), suggesting that the colour effects of chitosan amount are storage-time dependent. The AC × CC interaction was significant for all six-colour parameters: L^* ($p < 0.05$), a^* ($p < 0.001$), b^* ($p < 0.05$), C^* ($p < 0.05$), h ($p < 0.001$) and λ ($p < 0.001$), highlighting a strong synergistic effect between chitosan amount and coating presence. The ST × P interaction was also significant for L^* ($p < 0.01$), a^* ($p < 0.001$), C^* ($p < 0.01$), h ($p < 0.001$) and λ ($p < 0.01$), however not for b^* ($P > 0.05$), pointing to the importance of packaging choices over time. The AC × P interaction significantly influenced a^* ($p < 0.05$), b^* ($p < 0.05$), h ($p < 0.01$) and λ ($p < 0.01$), but not L^* ($P > 0.05$) and C^* ($P > 0.05$). The CC × P interaction was significant only for h ($p < 0.05$). Random effects and their interactions were mostly not significant. The repetition term (RP) had P values above 0.270 for all parameters. However, RP × AC was significant for b^* ($p < 0.05$) and C^* ($p < 0.05$), indicating some variability among replicates. All other random interaction terms (e.g., RP × ST, RP × CC, RP × P) showed non-significance ($P > 0.05$ for all colour parameters) (Table S5).

Table S5. ANOVA analysis for cut cross section colour characteristics of tea sausage.

	**Effect**	***L****	***a****	***b****	***C****	***h***	**λ**
Intercept	Fixed	< 0.001	< 0.001	< 0.001	< 0.001	< 0.001	< 0.001
RP	Random	0.270	0.623	0.935	0.591	0.989	0.527
ST	Fixed	< 0.001	< 0.001	0.930	< 0.001	< 0.001	< 0.001
AC	Fixed	0.023	0.002	0.091	0.001	0.537	0.246
CC	Fixed	0.492	0.011	0.066	0.009	0.423	0.700
P	Fixed	< 0.001	< 0.001	< 0.001	< 0.001	0.790	0.461
RP × ST	Random	0.083	0.214	0.553	0.310	0.539	0.232
RP × AC	Random	0.443	0.425	0.014	0.049	0.953	0.954
ST × AC	Fixed	0.657	0.024	0.262	0.097	0.001	0.002
RP × CC	Random	0.126	0.724	0.903	0.651	0.991	0.990
ST × CC	Fixed	0.802	0.087	0.768	0.186	0.143	0.519
AC × CC	Fixed	0.031	< 0.001	0.039	0.031	< 0.001	< 0.001
RP × P	Random	0.447	0.687	0.305	0.437	0.783	0.772
ST × P	Fixed	0.001	< 0.001	0.752	0.002	< 0.001	0.001
AC × P	Fixed	0.083	0.048	0.036	0.247	0.001	0.003
CC × P	Fixed	0.428	0.157	0.517	0.435	0.047	0.172

AC – amount of chitosan; CC – chitosan coating; P – packaging; ST – storage time; RP – repetitions.

ANOVA analysis – Texture characteristics of tea sausage

The ANOVA analysis of texture stability parameters – hardness, springiness, cohesiveness and chewiness – demonstrates significant influences of both individual and interactive effects of formulation and storage factors. The amount of chitosan (AC) showed a highly significant impact on all four texture parameters ($p < 0.001$), confirming its critical role in maintaining structural integrity. Similarly, the chitosan coating (CC) significantly affected hardness, springiness and cohesiveness ($p < 0.001$ for each), but not chewiness ($P > 0.05$), whereas packaging (P) significantly influenced hardness ($p < 0.001$), cohesiveness ($p < 0.01$) and chewiness ($p < 0.05$), but not springiness ($P > 0.05$). Storage time (ST) had a significant effect on springiness ($p < 0.001$), cohesiveness ($p < 0.001$) and chewiness ($p < 0.01$), but not on hardness ($P > 0.05$), indicating that prolonged storage primarily impacts elastic and cohesive properties. Among interaction effects, ST × AC was highly significant across all parameters ($p < 0.05$), especially for hardness, cohesiveness and chewiness (all $p < 0.001$), suggesting that the influence of chitosan concentration on texture depends strongly on storage duration. The ST × CC interaction was also significant for hardness and chewiness ($p < 0.05$), while AC × CC had a marginal effect on hardness ($p < 0.05$). Notably, AC × P significantly affected hardness ($p < 0.01$), springiness ($p < 0.05$) and chewiness ($p < 0.001$), and CC × P was significant for hardness ($p < 0.05$) and chewiness ($p < 0.05$), emphasizing the combinatory role of chitosan and packaging. The ST × P interaction was significant only for chewiness ($p < 0.05$). Random effects were mostly not significant, except for repetitions (RP) in cohesiveness ($p < 0.05$), indicating limited variability due to assessor differences. Collectively, these findings highlight that chitosan-related factors, particularly in interaction with storage time and packaging, are central to preserving textural attributes, with AC being the most consistently influential variable (Table S6).

Table S6. ANOVA analysis for texture characteristics of tea sausage.

	Effect	Hardness	Springiness	Cohesiveness	Chewiness
Intercept	Fixed	< 0.001	< 0.001	< 0.001	< 0.001
RP	Random	0.799	0.626	0.049	0.991
ST	Fixed	0.417	< 0.001	< 0.001	0.005
AC	Fixed	< 0.001	< 0.001	< 0.001	< 0.001
CC	Fixed	< 0.001	< 0.001	< 0.001	0.550
P	Fixed	< 0.001	0.878	0.002	0.010
RP × ST	Random	0.723	0.217	0.462	0.822
RP × AC	Random	0.827	0.791	0.821	0.825
ST × AC	Fixed	< 0.001	0.042	< 0.001	< 0.001
RP × CC	Random	0.975	0.707	0.754	0.986
ST × CC	Fixed	0.004	0.371	0.894	0.004
AC × CC	Fixed	0.045	0.389	0.237	0.117
RP × P	Random	0.917	0.850	0.669	0.876
ST × P	Fixed	0.250	0.305	0.309	0.022
AC × P	Fixed	0.003	0.010	0.617	< 0.001
CC × P	Fixed	0.021	0.413	0.828	0.016

AC – amount of chitosan; CC – chitosan coating; P – packaging; ST – storage time; RP – repetitions.

ANOVA analysis – Overall sensory quality of tea sausage

The ANOVA results for sensory stability demonstrate that all main fixed effects – storage time (ST), amount of chitosan (AC), chitosan coating (CC) and packaging (P) – significantly influenced sensory stability ($p < 0.01$), confirming their independent contributions to sensory quality. Among the fixed interaction effects, significant interactions were found for ST × CC ($p < 0.01$), AC × CC ($p < 0.001$) and AC × P ($p < 0.05$), indicating that the sensory perception was modulated by the combined effects of chitosan-related treatments and packaging. In contrast, interactions such as ST × AC, ST × P and CC × P were not significant ($P > 0.05$), suggesting minimal joint effects among those factors. Furthermore, random effects including RP ($p < 0.001$), RP × ST ($p < 0.001$) and RP × CC ($p < 0.01$) were statistically significant, pointing to notable variability in sensory evaluations across repetitions and interactions with specific treatment factors. These findings underscore the critical roles of both individual treatments and their combinations, particularly those involving chitosan and storage, in shaping sensory stability, while also highlighting the importance of accounting for assessor variability (Table S7).

Table S7. ANOVA analysis for overall sensory quality of tea sausage.

	**Effect**	**Sensory analysis**
Intercept	Fixed	< 0.001
RP	Random	< 0.001
ST	Fixed	< 0.001
AC	Fixed	0.001
CC	Fixed	< 0.001
P	Fixed	0.002
RP × ST	Random	< 0.001
RP × AC	Random	0.234
ST × AC	Fixed	0.721
RP × CC	Random	0.002
ST × CC	Fixed	0.001
AC × CC	Fixed	< 0.001
RP × P	Random	0.269
ST × P	Fixed	0.586
AC × P	Fixed	0.036
CC × P	Fixed	0.843

AC – amount of chitosan; CC – chitosan coating; P – packaging; ST – storage time; RP – repetitions.

ANOVA analysis – Microbiological characteristics of tea sausage

The univariate ANOVA for microbiological characteristics, specifically TPC and LAB, revealed that storage time (ST) had a highly significant effect on both parameters ($p < 0.001$), confirming its critical role in microbial dynamics. The amount of chitosan (AC) significantly affected TPC ($p < 0.01$), but not LAB ($P > 0.05$), while chitosan coating (CC) showed marginal significance for both TPC ($P > 0.05$) and LAB ($P > 0.05$), suggesting a moderate antimicrobial effect. Packaging (P) alone did not significantly influence either microbial parameter ($P > 0.05$). However, several two-way interactions revealed important synergies: ST × AC, ST × CC and ST × P were all highly significant for both TPC and LAB ($p < 0.05$), indicating that the effectiveness of chitosan and packaging interventions is strongly modulated by storage time. Additionally, AC × CC showed strong significance (TPC: $p < 0.01$; LAB: $p < 0.01$) and AC × P significantly influenced TPC ($p < 0.05$), highlighting combinatory effects between chitosan formulation and packaging. In contrast, all random effects and their interactions were non-significant ($P > 0.05$), indicating minimal variability due to repetitions. These results emphasize the dominant role of storage time and the interactive influence of chitosan treatments and packaging on the microbiological stability of the product (Table S8).

Table S8. ANOVA analysis for microbiological characteristics of tea sausage.

	Effect	TPC	LAB
Intercept	Fixed	< 0.001	< 0.001
RP	Random	0.912	1.000
ST	Fixed	< 0.001	< 0.001
AC	Fixed	0.009	0.257
CC	Fixed	0.081	0.054
P	Fixed	0.520	0.386
RP × ST	Random	0.961	0.996
RP × AC	Random	0.979	1.000
ST × AC	Fixed	< 0.001	< 0.001
RP × CC	Random	0.864	1.000
ST × CC	Fixed	< 0.001	0.047
AC × CC	Fixed	0.003	0.001
RP × P	Random	0.562	0.999
ST × P	Fixed	< 0.001	< 0.001
AC × P	Fixed	0.024	0.741
CC × P	Fixed	0.156	0.469

TPC – total plate count; LAB – lactic acid bacteria; AC – amount of chitosan; CC – chitosan coating; P – packaging; ST – storage time; RP – repetitions.

Table S9. The effect of two-way. three-way and four-way interactions among processing parameters on the quality of tea sausages (dry fermented sausages) expressed as *p* value.

***p* value**	**pH**	**Surface**						**Cut cross section**					
		L*	***a****	***b****	***C****	***h***	***λ***	***L****	***a****	***b****	***C****	***h***	***λ***
AC x CC	< 0.001	0.057	0.002	0.116	0.198	< 0.001	< 0.001	0.038	< 0.001	0.053	0.025	< 0.001	< 0.001
AC x P	0.006	0.033	0.072	0.667	0.167	0.140	0.069	0.138	0.048	0.061	0.270	< 0.001	0.002
AC x ST	0.117	0.028	0.843	0.436	0.976	0.081	0.039	0.586	0.013	0.339	0.066	0.001	0.001
CC x P	0.087	0.255	< 0.001	0.149	< 0.001	< 0.001	< 0.001	0.319	0.234	0.387	0.621	0.031	0.164
CC x ST	0.905	0.010	0.005	0.060	0.007	0.440	0.061	0.873	0.133	0.993	0.313	0.093	0.479
P x ST	0.905	0.522	0.009	0.713	0.102	< 0.001	< 0.001	0.001	< 0.001	0.633	0.003	< 0.001	< 0.001
AC x CC x P	0.442	0.930	0.620	0.372	0.472	0.695	0.589	0.124	< 0.001	0.621	0.005	< 0.001	< 0.001
AC x CC x ST	0.534	0.528	0.388	0.717	0.659	0.090	0.055	0.669	0.001	0.008	0.129	< 0.001	< 0.001
AC x P x ST	0.128	0.362	0.019	0.310	0.096	< 0.001	0.001	0.294	0.008	0.004	0.011	< 0.001	0.001
CC x P x ST	0.511	0.082	0.673	0.646	0.553	0.534	0.837	0.049	0.187	0.620	0.352	0.161	0.857
AC x CC x P x ST	0.138	0.952	< 0.001	0.036	< 0.001	< 0.001	< 0.001	0.610	< 0.001	0.473	< 0.001	< 0.001	0.012

AC – amount of chitosan; CC – chitosan coating; P – packaging; ST – storage time.

Table S10. The effect of two-way. three-way and four-way interactions among processing parameters on the quality of tea sausages (dry fermented sausages) expressed as *p* value (continued).

***p* value**	**Hardness**	**Springiness**	**Cohesiveness**	**Chewiness**	**Overall sensory quality**	**TPC**	**LAB**
AC x CC	0.030	0.352	0.262	0.082	0.004	0.001	< 0.001
AC x P	0.001	0.007	0.604	< 0.001	0.290	0.011	0.676
AC x ST	< 0.001	0.037	< 0.001	< 0.001	0.886	< 0.001	< 0.001
CC x P	0.015	0.383	0.859	0.009	0.904	0.120	0.407
CC x ST	0.003	0.379	0.880	0.002	0.038	< 0.001	0.023
P x ST	0.231	0.316	0.287	0.015	0.741	< 0.001	< 0.001
AC x CC x P	< 0.001	0.093	0.744	< 0.001	0.635	0.102	0.049
AC x CC x ST	0.006	< 0.001	0.123	< 0.001	0.747	< 0.001	0.001
AC x P x ST	< 0.001	0.287	0.493	< 0.001	0.746	0.033	0.001
CC x P x ST	< 0.001	0.792	0.039	< 0.001	0.496	0.001	< 0.001
AC x CC x P x ST	0.307	0.705	0.114	0.047	0.470	< 0.001	< 0.001

AC – amount of chitosan; CC – chitosan coating; P – packaging; ST – storage time.

pH value and surface colour characteristics of tea sausage

Chitosan coating and storage time had no significant effect ($P > 0.05$) on the pH values, while amount of chitosan and packaging had significant effect ($p < 0.05$) on the pH values. Tea sausages produced with 0% of chitosan had the lowest pH values, while tea sausages produced with 0.50% of chitosan had the highest pH values. pH values in vacuum packed sausages were lower than in MAP packed sausages (Table S10). The two-way (AC x CC; AC x P) interaction had a significant (p = 0.006 – < 0.001) effect on the pH values (Table S9).

Amount of chitosan had no significant effect ($p > 0.05$) on the surface L^* values, while chitosan coating, packaging and storage time had significant effect ($p < 0.05$) on the surface L^* values. Chitosan coating had the effect of increasing surface L^* values. Surface L^* values in MAP packed sausages were lower than in unpacked. Concerning storage time, decreasing of surface L^* values on the 90th day of storage can be noticed. Amount of chitosan had no significant effect ($p > 0.05$) on the surface a^* values, while chitosan coating, packaging and storage time had significant ($p < 0.05$) effect on the surface a^* values. Surface a^* values on uncoated sausages were higher than on chitosan coated sausages. Also, the highest surface a^* values were recorded in unpacked sausages. Moreover, storage time had the effect of decreasing surface a^* values. Amount of chitosan, chitosan coating, packaging and storage time had significant ($p < 0.05$) effect on the surface b^*, C^*, h and λ values. Tea sausages produced with 0% of chitosan had the highest surface b^* values, while tea sausages produced with 0.50% of chitosan had the lowest surface b^* values. For the other treatments that show significant effects, surface b^* values show the same decreasing trend as surface a^* values. Tea sausages produced with 0 and 0.25% of chitosan had higher surface C^* values than tea sausages produced with 0.50% of chitosan. For the other treatments that show significant effects, surface C^* values show the same decreasing trend as surface a^* and b^* values. Tea sausages produced without chitosan had higher surface h and lower surface λ values than tea sausages produced with chitosan (0.25 and 0.50%). Chitosan coating had the effect of increasing surface h values (same trend as for L^* value). Also, the lowest h values were recorded in unpacked sausages. Concerning storage time, the increasing of surface h values after the 45th day of storage can be noticed, and that stayed at the same level. For the other treatments that show significant effects, surface λ values show the same decreasing trend as surface a^*, b^* and C^* values (Table S10). Although statistical differences existed for surface colour characteristics, sometimes numerical differences were not of great magnitude. The two-way (AC x P; AC x ST; CC x ST) interaction had a significant (p = 0.033 – 0.010) effect on the surface L^* values; two-way (AC x CC; CC x P; CC x ST; P x ST) and four-way (AC x CC x P x ST) interactions had significant (p = 0.009 – < 0.001) effect on the surface a^* values; four-way (AC x CC x P x ST) interaction had a significant (P = 0.036) effect on the surface b^* values; two-way (CC x P; CC x ST) and four-way (AC x CC x P x ST) interactions had significant (p = 0.007 – < 0.001) effect on the surface C^* values; two-way (AC x CC; CC x P; P x ST), three-way (AC x P x ST) and four-way (AC x CC x P x ST) interactions had significant ($p < 0.001$) effect on the surface h values; two-way (AC x CC; AC x ST; CC x P; P x ST), three-way (AC x P x ST) and four-way (AC x CC x P x ST) interactions had significant (P = 0.039 – < 0.001) effect on the surface λ values (Table S9).

Table S11. pH value and surface colour characteristics of tea sausage.

**Treatments**	**pH**	***L****	***a****	***b****	***C****	***h***	**λ (nm)**
Effect of amount of chitosan (AC)							
0%	5.36±0.02[c]	30.2±0.23	13.4±0.31	10.6±0.12[a]	17.1±0.29[a]	38.9±0.57[a]	595.7±0.36[b]
0.25%	5.47±0.01[b]	30.1±0.20	13.5±0.24	9.9±0.14[b]	16.8±0.27[a]	36.4±0.27[b]	597.0±0.21[a]
0.50%	5.57±0.01[a]	30.0±0.18	12.8±0.28	9.4±0.15[c]	16.0±0.30[b]	36.7±0.36[b]	596.7±0.26[a]
p value	< 0.001	0.638	0.177	< 0.001	0.013	< 0.001	0.005
Effect of chitosan coating (CC)							
Uncoated	5.48±0.02	29.4±0.15[b]	15.0±0.14[a]	10.6±0.10[a]	18.4±0.16[a]	35.3±0.15[b]	598.1±0.11[a]
Coated	5.45±0.02	31.0±0.15[a]	11.1±0.18[b]	9.2±0.10[b]	14.5±0.18[b]	39.9±0.43[a]	594.4±0.24[b]
p value	0.176	< 0.001	< 0.001	< 0.001	< 0.001	< 0.001	< 0.001
Effect of packaging (P)							
Unpacked	5.47±0.01[ab]	31.0±0.14[a]	15.9±0.10[a]	11.0±0.10[a]	19.3±0.14[a]	34.7±0.12[b]	598.6±0.07[a]
V	5.52±0.02[a]	30.3±0.16[ab]	12.9±0.22[b]	9.7±0.12[b]	16.2±0.24[b]	37.3±0.35[a]	596.3±0.23[b]
MAP	5.41±0.02[b]	29.7±0.19[b]	13.0±0.25[b]	10.0±0.12[b]	16.5±0.25[b]	38.0±0.42[a]	596.1±0.27[b]
p value	< 0.001	0.001	< 0.001	< 0.001	< 0.001	< 0.001	< 0.001
Effect of storage time (days) (ST)							
0	5.47±0.01	31.0±0.28[a]	15.9±0.20[a]	11.0±0.21[a]	19.3±0.28[a]	34.7±0.25[b]	598.6±0.14[a]
45	5.48±0.02	30.3±0.19[a]	13.6±0.25[b]	10.1±0.13[b]	17.0±0.26[b]	37.1±0.39[a]	596.7±0.26[b]
90	5.45±0.02	29.6±0.16[b]	12.3±0.21[c]	9.6±0.12[c]	15.6±0.22[c]	38.3±0.38[a]	595.7±0.24[c]
p value	0.407	< 0.001	< 0.001	< 0.001	< 0.001	< 0.001	< 0.001

Results are reported as means ± standard errors of the mean. V – vacuum; MAP – modified atmosphere packaging; *L** – a measure of darkness/lightness (higher value indicates a lighter colour); *a** – a measure of redness (higher value indicates a redder colour); *b** – a measure of yellowness (higher value indicates a more yellow colour); *h* – hue angle (lower values indicates a redder colour); *C** – saturation index (higher values indicates greater saturation of red); *λ* – dominant wavelength (orange colour: 585-620 nm); [abc] $p < 0.05$.

Cut cross section colour characteristics of tea sausage

Amount of chitosan had no significant effect ($p > 0.05$) on the cut cross section L^* values, while chitosan coating, packaging and storage time had significant effect ($p < 0.05$) on the cut cross section L^* values. Chitosan coating had the effect of decreasing cut cross section L^* values. Unpacked tea sausages had the highest cut cross section L^* values, while tea sausages packed in vacuum had the lowest cut cross section L^* values. Moreover, storage time had the effect of decreasing cut cross section L^* values. Chitosan coating had no significant effect ($P > 0.05$) on the cut cross section a^* values, while amount of chitosan, packaging and storage time had significant effect ($p < 0.05$) on the cut cross section a^* values. Tea sausages produced with 0 and 0.25% of chitosan had higher cut cross section a^* values than tea sausages produced with 0.50% of chitosan. For the other treatments that show significant effects, cut cross section a^* values show the same numerical changing trend as cut cross section L^* values. Amount of chitosan and chitosan coating had no significant effect ($p > 0.05$) on the cut cross section b^* and C^* values, while packaging and storage time had significant effect ($p < 0.05$) on the cut cross section b^* and C^* values. Cut cross section b^* values in unpacked and MAP packed sausages were higher than in vacuum packed. Concerning storage time, decreasing of cut cross section b^* values up to the 45th day of storage can be noticed, and that stayed at the same level. For the packaging and storage time treatment, cut cross section C^* values show the same numerical changing trend as cut cross section L^* and a^* values. Chitosan coating and packaging had no significant effect ($p > 0.05$) on the cut cross section h and λ values, while amount of chitosan and storage time had significant effect ($p < 0.05$) on the cut cross section h and λ values. Tea sausages produced with 0 and 0.25% of chitosan had lower cut cross section h and higher cut cross section λ values than tea sausages produced with 0.50% of chitosan. Concerning storage time, the increasing of cut cross section h values on the 90th day of storage can be noticed. Cut cross section λ values were the highest after 45 days of storage and the lowest after the 90 days of storage (Table S11). Although statistical differences existed for cut cross section colour characteristics, sometimes numerical differences were not of great magnitude. The two-way (AC x CC; P x ST) and three-way (CC x P x ST) interactions had significant ($p = 0.049 – 0.001$) effect on the cut cross section L^* values; two-way (AC x CC; AC x P; AC x ST; P x ST), three-way (AC x CC x P; AC x CC x ST; AC x P x ST) and four-way (AC x CC x P x ST) interactions had significant ($p = 0.048 – < 0.001$) effect on the cut cross section a^* values; three-way (AC x CC x ST; AC x P x ST) interaction had a significant ($p = 0.008 – 0.004$) effect on the cut cross section b^* values; two-way (AC x CC; P x ST), three-way (AC x CC x P; AC x P x ST) and four-way (AC x CC x P x ST) interactions had significant ($p = 0.025 – < 0.001$) effect on the cut cross section C^* values; two-way (AC x CC; AC x P; AC x ST; CC x P; P x ST), three-way (AC x CC x P; AC x CC x ST; AC x P x ST) and four-way (AC x CC x P x ST) interactions had significant ($p = 0.031 – < 0.001$) effect on the cut cross section h values; two-way (AC x CC; AC x P; AC x ST; P x ST), three-way (AC x CC x P; AC x CC x ST; AC x P x ST) and four-way (AC x CC x P x ST) interactions had significant ($p = 0.012 – < 0.001$) effect on the cut cross section λ values (Table S9).

Table S12. Cut cross section colour characteristics of tea sausage.

Treatments	*L**	*a**	*b**	*C**	*h*	λ (nm)
Effect of amount of chitosan (AC)						
0%	41.0±0.27	14.0±0.14[a]	6.8±0.08	15.6±0.15	25.9±0.25[b]	605.4±0.29[a]
0.25%	41.6±0.26	13.9±0.11[a]	6.9±0.08	15.6±0.13	26.3±0.17[b]	604.6±0.21[a]
0.50%	40.7±0.27	13.4±0.19[b]	7.0±0.09	15.2±0.17	28.1±0.61[a]	603.5±0.40[b]
p value	0.051	0.012	0.127	0.165	< 0.001	< 0.001
Effect of chitosan coating (CC)						
Uncoated	41.5±0.21[a]	13.8±0.13	6.9±0.06	15.5±0.12	27.0±0.37	604.4±0.26
Coated	40.5±0.22[b]	13.8±0.12	6.9±0.07	15.4±0.13	26.5±0.21	604.6±0.24
p value	0.001	0.991	0.501	0.708	0.301	0.571
Effect of packaging (P)						
Unpacked	45.1±0.30[a]	14.8±0.21[a]	7.3±0.13[a]	16.5±0.24[a]	26.4±0.25	604.5±0.33
V	40.0±0.22[c]	13.0±0.16[c]	6.5±0.07[b]	14.6±0.13[c]	27.0±0.51	604.6±0.37
MAP	40.9±0.18[b]	14.3±0.09[b]	7.2±0.06[a]	16.0±0.10[b]	26.6±0.15	604.4±0.17
p value	< 0.001	< 0.001	< 0.001	< 0.001	0.586	0.917
Effect of storage time (days) (ST)						
0	45.1±0.30[a]	14.8±0.21[a]	7.3±0.13[a]	16.5±0.24[a]	26.4±0.25[b]	604.5±0.33[b]
45	42.0±0.17[b]	14.2±0.10[b]	6.8±0.07[b]	15.8±0.12[b]	25.5±0.15[b]	605.7±0.18[a]
90	38.8±0.18[c]	13.1±0.15[c]	6.9±0.07[b]	14.9±0.13[c]	28.2±0.50[a]	603.3±0.34[c]
p value	< 0.001	< 0.001	< 0.001	< 0.001	< 0.001	

Results are reported as means ± standard errors of the mean. V – vacuum; MAP – modified atmosphere packaging; *L** – a measure of darkness/lightness (higher value indicates a lighter colour); *a** – a measure of redness (higher value indicates a redder colour); *b** – a measure of yellowness (higher value indicates a more yellow colour); *h* – hue angle (lower values indicates a redder colour); *C** – saturation index (higher values indicates greater saturation of red); λ – dominant wavelength (orange colour: 585-620 nm); [abc] $p < 0.05$.

Texture characteristics of tea sausage

Amount of chitosan, chitosan coating, packaging and storage time had significant ($p < 0.05$) effect on the hardness, springiness, cohesiveness and chewiness values, except chitosan coating on the chewiness. Tea sausages produced with 0 and 0.25% of chitosan had higher hardness values than tea sausages produced with 0.50% of chitosan. Also, the addition of chitosan decreased the springiness, cohesiveness and chewiness values. Chitosan coating had the effect of decreasing hardness values and increasing springiness and cohesiveness values. Unpacked tea sausages had the lowest hardness values, while tea sausages packed in MAP had the highest hardness values. Springiness and chewiness values in unpacked sausages were lower than in packed (V and MAP), while cohesiveness values in packed (V and MAP) sausages were lower than in unpacked. Concerning storage time, the increasing of hardness and chewiness values up to the 45th day of storage can be noticed, and that stayed at the same level, while the increasing of springiness values after the 90th day of storage can be noticed. Storage time had the effect of decreasing cohesiveness values (Table S12). Although statistical differences existed for springiness and cohesiveness, numerical differences were not of great magnitude. The two-way (AC x CC; AC x P; AC x ST; CC x P; CC x ST) and three-way (AC x CC x P; AC x CC x ST; AC x P x ST; CC x P x ST) interactions had significant (p = 0.030 – < 0.001) effect on the hardness values; two-way (AC x P; AC x ST) and three-way (AC x CC x ST) interactions had significant (P = 0.037 – < 0.001) effect on the springiness values; two-way (AC x ST) and three-way (CC x P x ST) interactions had significant (p = 0.039 – < 0.001) effect on the cohesiveness values; two-way (AC x P; AC x ST; CC x P; CC x ST; P x ST), three-way (AC x CC x P; AC x CC x ST; AC x P x ST; CC x P x ST) and four-way (AC x CC x P x ST) interactions had significant ($p < 0.001$) effect on the chewiness values (Table S9).

Table S13. Texture characteristics of tea sausage.

Treatments	Hardness (g)	Springiness	Cohesiveness	Chewiness (g)
Effect of amount of chitosan (AC)				
0%	16761.6±292.5^{a}	0.459±0.004^{a}	0.467±0.003^{a}	3571.2±61.9^{a}
0.25%	16109.6±337.9^{a}	0.443±0.004^{b}	0.447±0.003^{b}	3181.3±72.0^{b}
0.50%	14003.0±265.1^{b}	0.428±0.004^{c}	0.430±0.003^{c}	2551.6±44.0^{c}
p value	< 0.001	< 0.001	< 0.001	< 0.001
Effect of chitosan coating (CC)				
Uncoated	16063.0±269.2^{a}	0.432±0.003^{b}	0.437±0.003^{b}	3028.1±56.1
Coated	15071.3±245.0^{b}	0.457±0.004^{a}	0.461±0.003^{a}	3193.9±66.8
p value	0.009	< 0.001	< 0.001	0.058
Effect of packaging (P)				
Unpacked	11176.9±292.4^{c}	0.423±0.004^{b}	0.469±0.005^{a}	2210.5±61.0^{b}
V	15687.2±231.8^{b}	0.447±0.004^{a}	0.449±0.003^{b}	3140.7±55.1^{a}
MAP	16675.8±259.5^{a}	0.446±0.004^{a}	0.441±0.003^{b}	3285.7±65.0^{a}
p value	< 0.001	0.010	< 0.001	< 0.001
Effect of storage time (days) (ST)				
0	11176.9±292.4^{b}	0.423±0.004^{b}	0.469±0.005^{a}	2210.5±61.0^{b}
45	16293.4±242.8^{a}	0.420±0.003^{b}	0.455±0.003^{b}	3131.5±61.8^{a}
90	16082.9±257.6^{a}	0.471±0.003^{a}	0.435±0.003^{c}	3296.1±58.7^{a}
p value	< 0.001	< 0.001	< 0.001	< 0.001

Results are reported as means ± standard errors of the mean. V – vacuum; MAP – modified atmosphere packaging; ^{abc} $p < 0.05$.

Overall sensory quality of tea sausage

Amount of chitosan, chitosan coating, packaging and storage time had significant ($p < 0.05$) effect on the overall sensory quality values. Tea sausages produced with chitosan (0.25 and 0.50%) had higher overall sensory scores than tea sausages produced without chitosan. Chitosan coating had the effect of decreasing overall sensory scores. Unpacked tea sausages had the lowest overall sensory scores, while tea sausages packed in vacuum had the highest (Table S13). The two-way (AC x CC; CC x ST) interaction had a significant ($p = 0.038 – 0.004$) effect on the overall sensory quality (Table S9).

Table S14. Overall sensory quality of tea sausage.

Treatments	Overall sensory quality
Effect of amount of chitosan (AC)	
0%	4.81±0.02^{b}
0.25%	4.87±0.02^{a}
0.50%	4.87±0.01^{a}
p value	0.019
Effect of chitosan coating (CC)	
Uncoated	4.88±0.01^{a}
Coated	4.82±0.02^{b}
p value	0.004
Effect of packaging (P)	
Unpacked	4.79±0.02^{b}
V	4.88±0.02^{a}
MAP	4.84±0.02^{ab}
P value	0.030
Effect of storage time (days) (ST)	
0	4.79±0.02^{b}
45	4.82±0.02^{b}
90	4.89±0.01^{a}
p value	< 0.001

Results are reported as means ± standard errors of the mean. V – vacuum; MAP – modified atmosphere packaging; ^{ab} $p < 0.05$.

Microbiological characteristics of tea sausage

Chitosan coating and packaging had no significant effect ($p > 0.05$) on the TPC values, while amount of chitosan and storage time had significant effect ($p < 0.05$) on the TPC values. In addition, amount of chitosan had no significant effect ($p > 0.05$) on the LAB values, while chitosan coating, packaging and storage time had significant effect ($p < 0.05$) on the LAB values. Tea sausages produced with 0 and 0.25% of chitosan had higher TPC values than tea sausages produced with 0.50% chitosan. Chitosan coating had the effect of decreasing LAB values. In contrast, the highest LAB values were recorded in unpacked sausages. Concerning storage time, decreasing of TPC values on the 90th day of storage can be noticed. Also, storage time had the effect of decreasing LAB values (Table S14). The two-way (AC x CC; AC x P; AC x ST; CC x ST; P x ST), three-way (AC x CC x ST; AC x P x ST; CC x P x ST) and four-way (AC x CC x P x ST) interactions had significant (p = 0.033 – < 0.001) effect on the TPC values; two-way (AC x CC; AC x ST; CC x ST; P x ST), three-way (AC x CC x P; AC x CC x ST; AC x P x ST; CC x P x ST) and four-way (AC x CC x P x ST) interactions had significant (p = 0.023 – < 0.001) effect on the LAB values (Table S9).

Table S15. Microbiological characteristics of tea sausage.

Treatments	TPC (log CFU/g)	LAB (log CFU/g)
Effect of amount of chitosan (AC)		
0%	4.16±0.06^{a}	7.06±0.09
0.25%	4.13±0.02^{a}	6.96±0.10
0.50%	4.00±0.05^{b}	7.05±0.08
p value	0.022	0.679
Effect of chitosan coating (CC)		
Uncoated	4.14±0.02	7.15±0.07^{a}
Coated	4.05±0.02	6.86±0.06^{b}
p value	0.064	0.005
Effect of packaging (P)		
Unpacked	4.27±0.04	7.91±0.05^{a}
V	4.06±0.04	6.89±0.09^{b}
MAP	4.09±0.03	6.93±0.05^{b}
p value	0.052	< 0.001
Effect of storage time (days) (ST)		
0	4.27±0.04^{a}	7.91±0.05^{a}
45	4.20±0.03^{a}	7.32±0.03^{b}
90	3.96±0.04^{b}	6.50±0.05^{c}
p value	< 0.001	< 0.001

Results are reported as means ± standard errors of the mean. V – vacuum; MAP – modified atmosphere packaging; TPC – total plate count; LAB – lactic acid bacteria; CFU – colony forming units; ^{abc} $p < 0.05$.

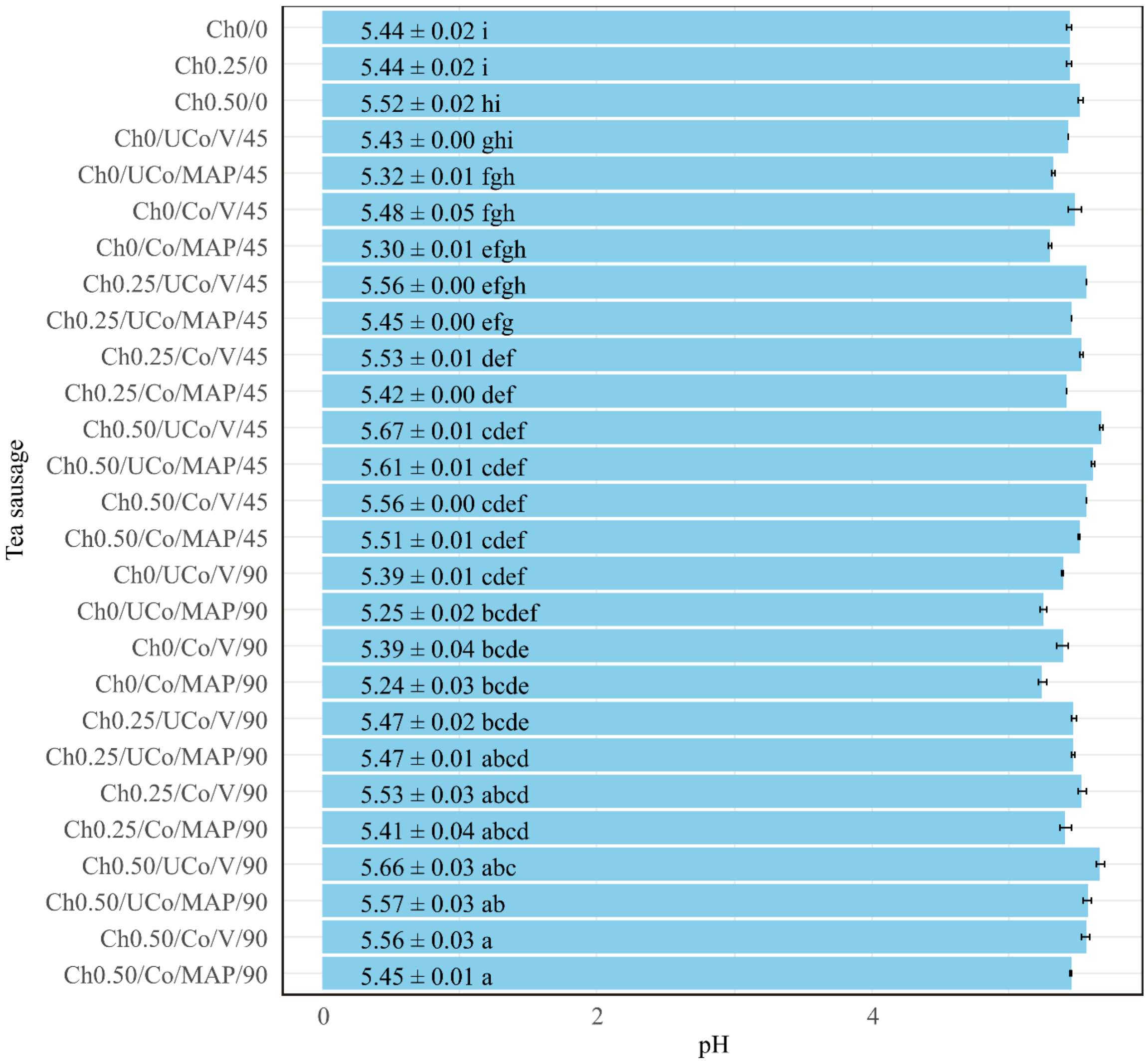

Figure S1. The effect of amount of chitosan, chitosan coating, packaging and storage time on pH values (mean ± SE) of tea sausages.
[abcdefghi] $p < 0.05$.

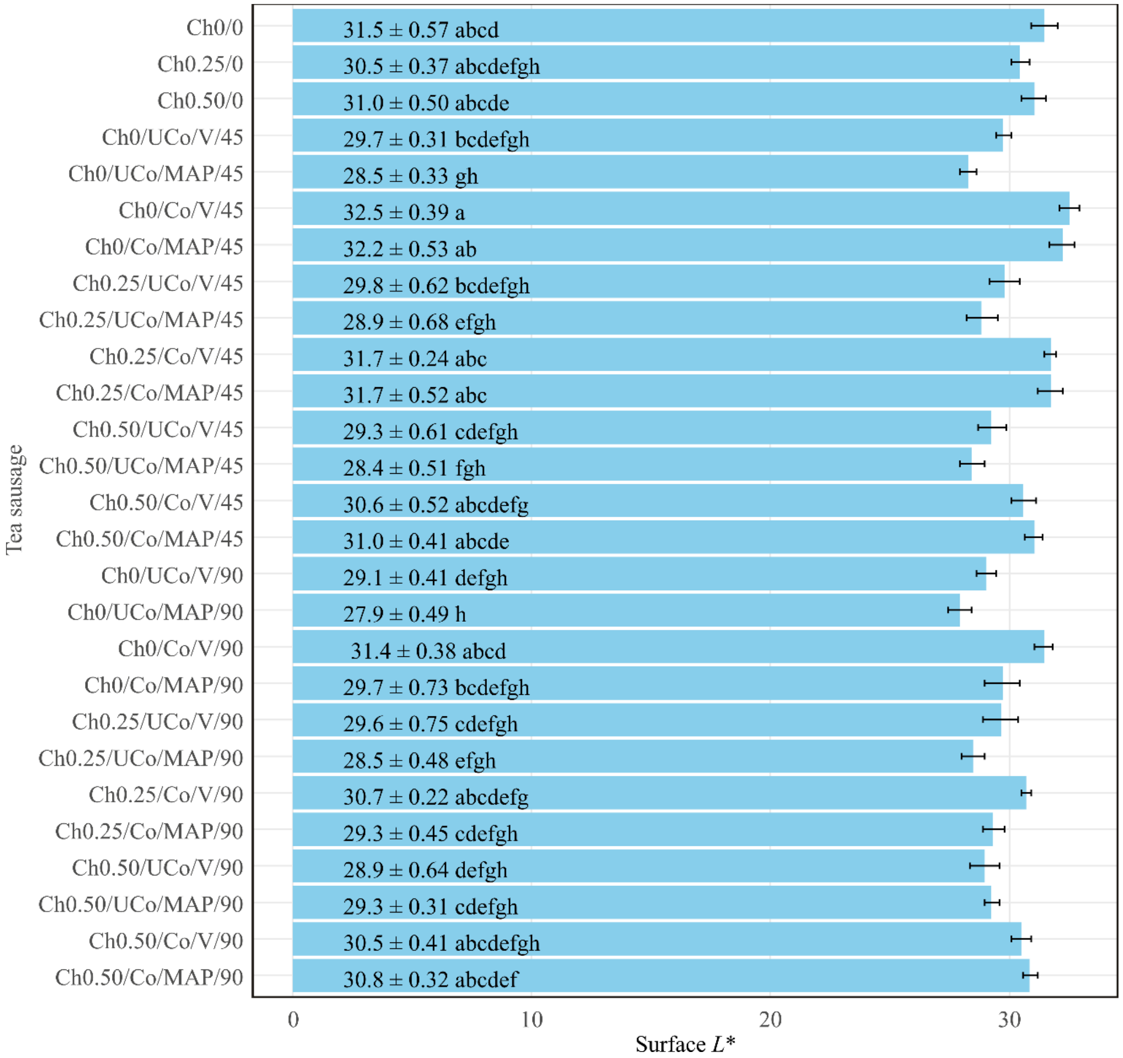

Figure S2. The effect of amount of chitosan, chitosan coating, packaging and storage time on surface L^* values (mean ± SE) of tea sausages. abcdefgh $p < 0.05$.

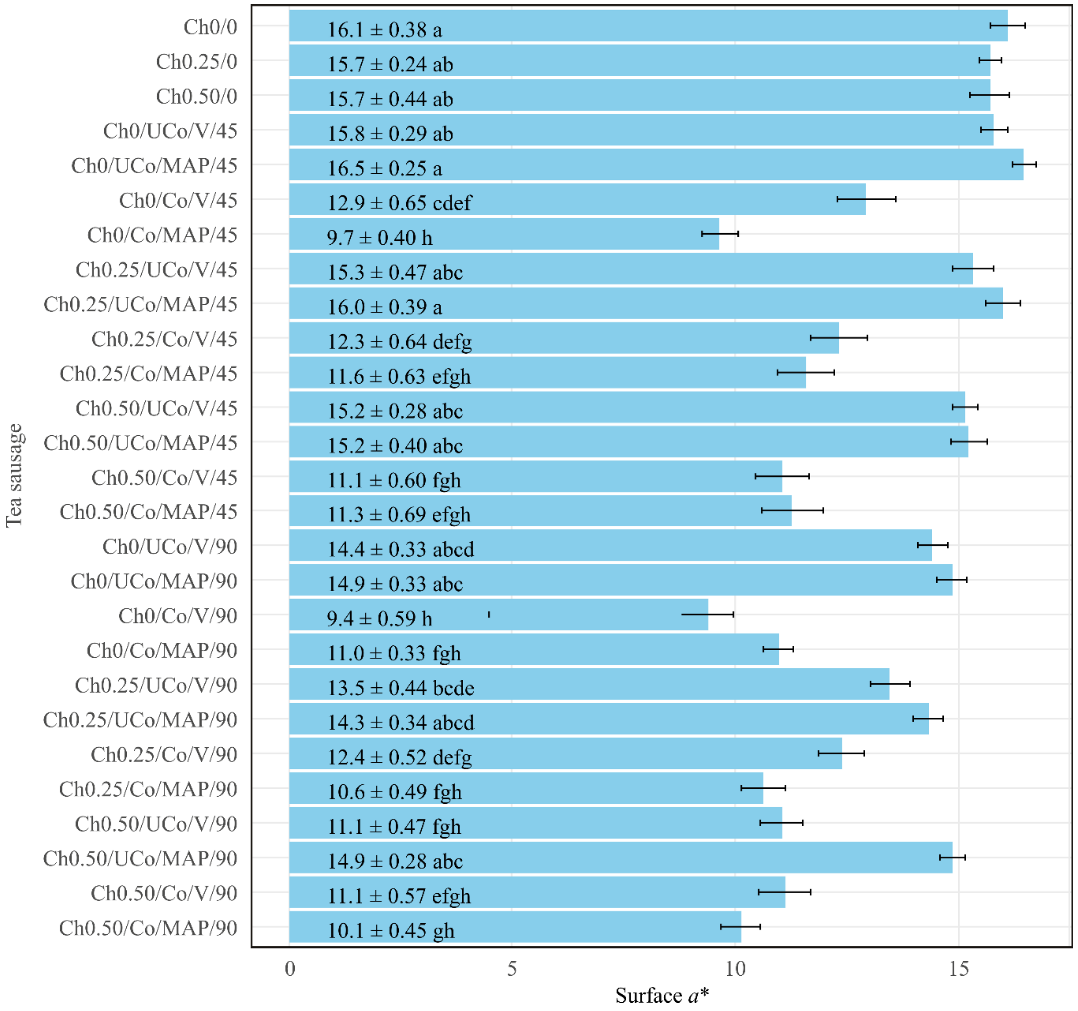

Figure S3. The effect of amount of chitosan, chitosan coating, packaging and storage time on surface a^* values (mean ± SE) of tea sausages. abcdefgh $p < 0.05$.

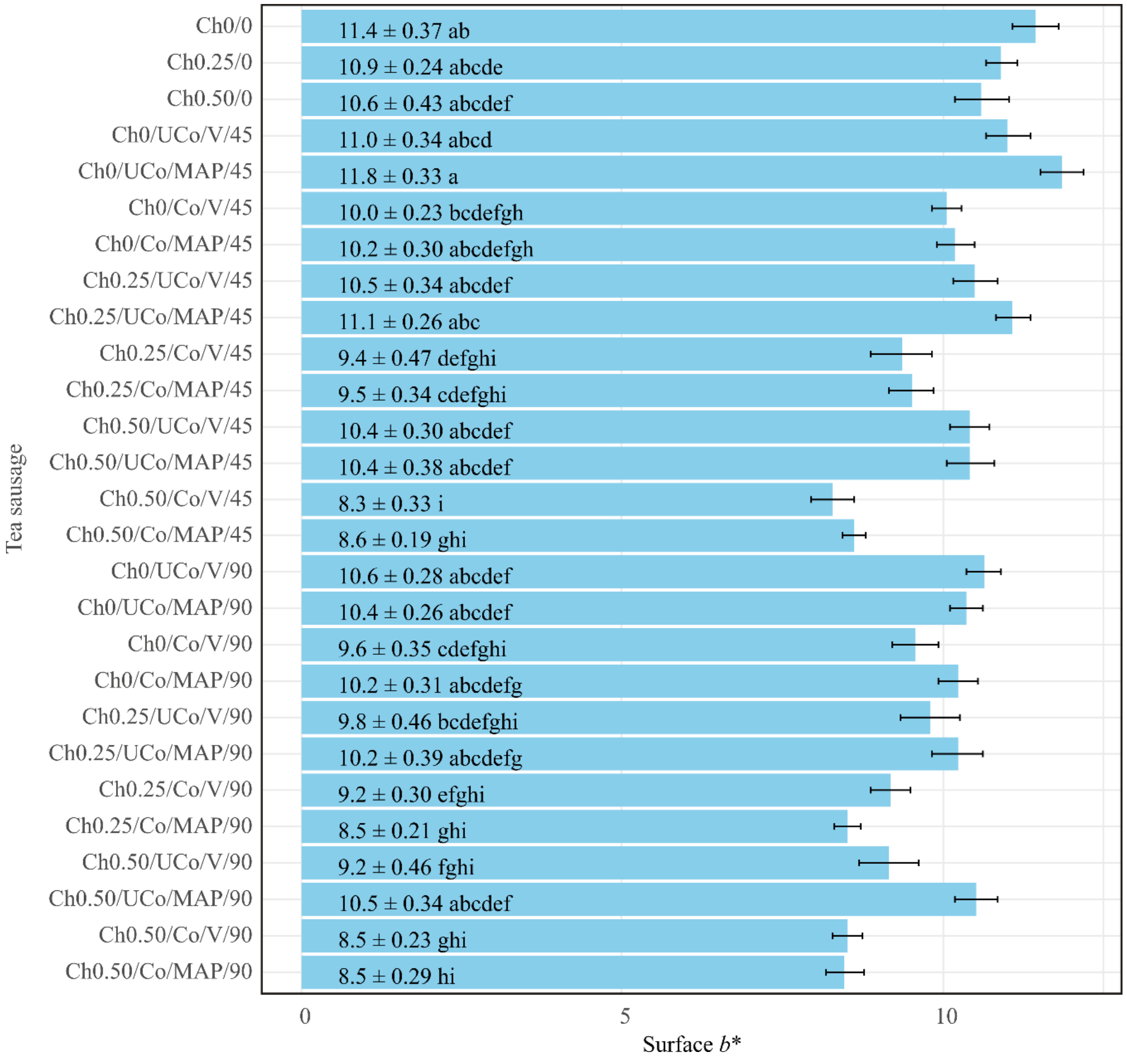

Figure S4. The effect of amount of chitosan, chitosan coating, packaging and storage time on surface b^* values (mean ± SE) of tea sausages. [abcdefghi] $p < 0.05$.

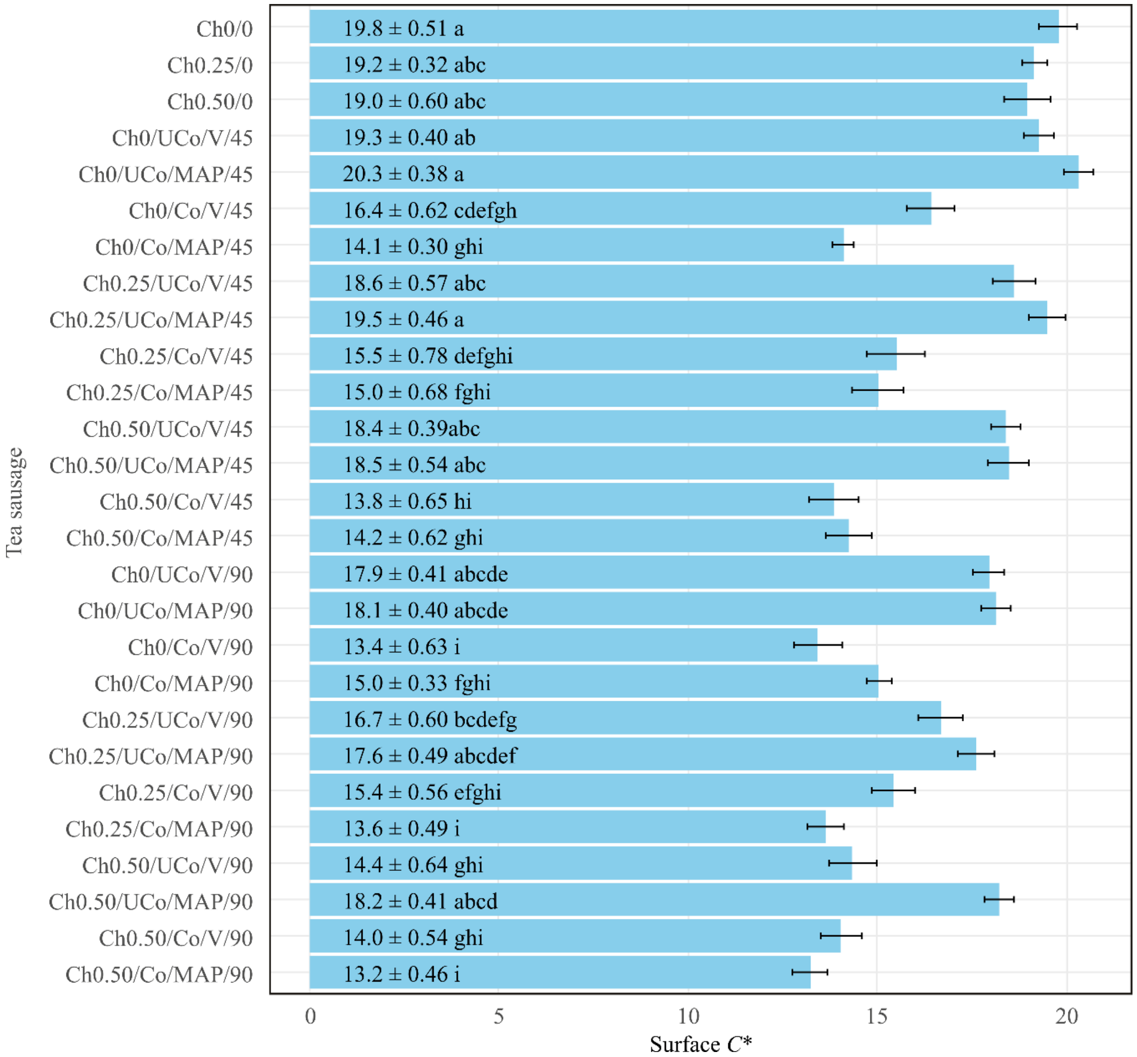

Figure S5. The effect of amount of chitosan, chitosan coating, packaging and storage time on surface C^* values (mean ± SE) of tea sausages. ^{abcdefghi} $p < 0.05$.

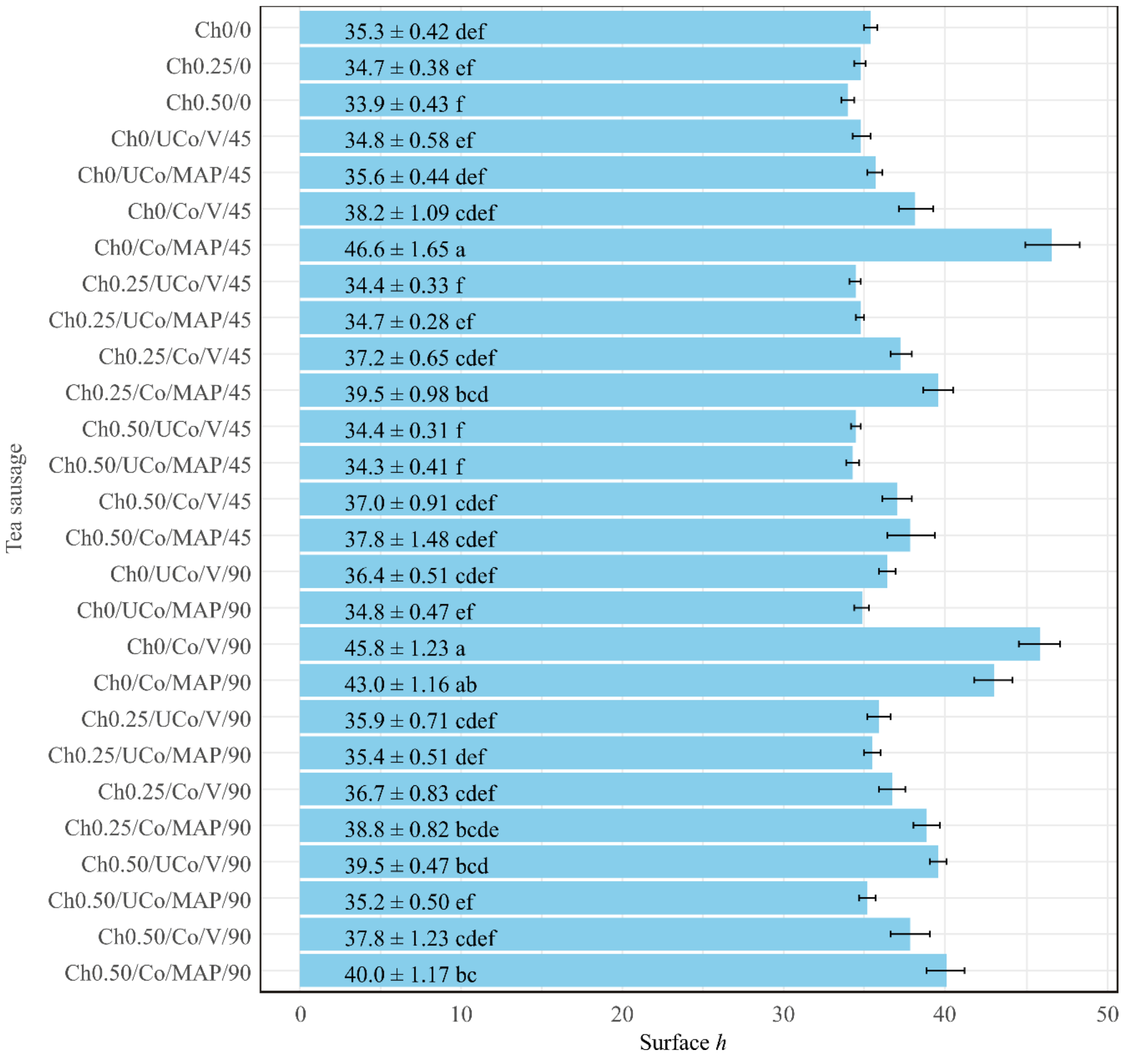

Figure S6. The effect of amount of chitosan, chitosan coating, packaging and storage time on surface h values (mean ± SE) of tea sausages. [abcdef] $p < 0.05$.

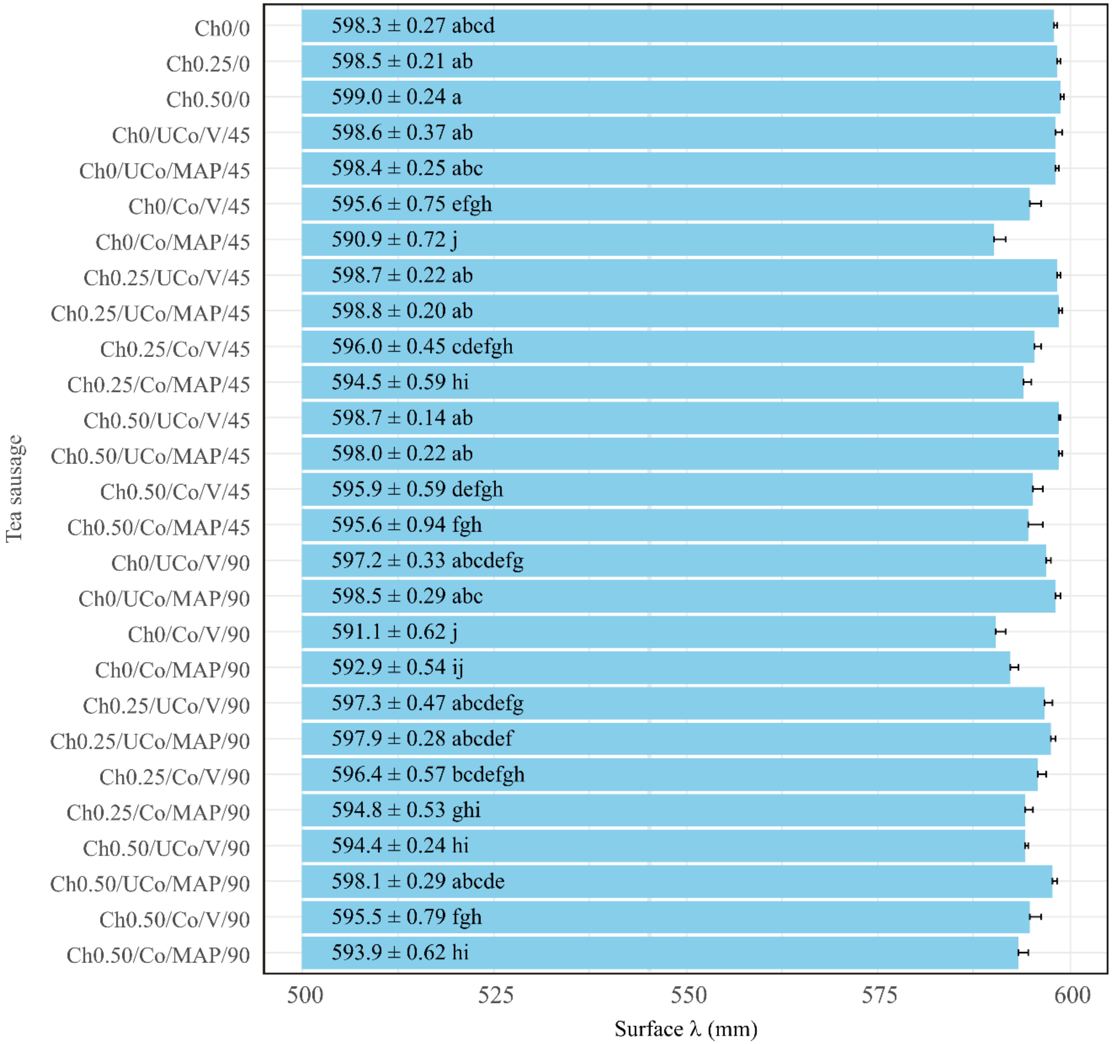

Figure S7. The effect of amount of chitosan, chitosan coating, packaging and storage time on surface *λ* values (mean ± SE) of tea sausages. [abcdefghij] $p < 0.05$.

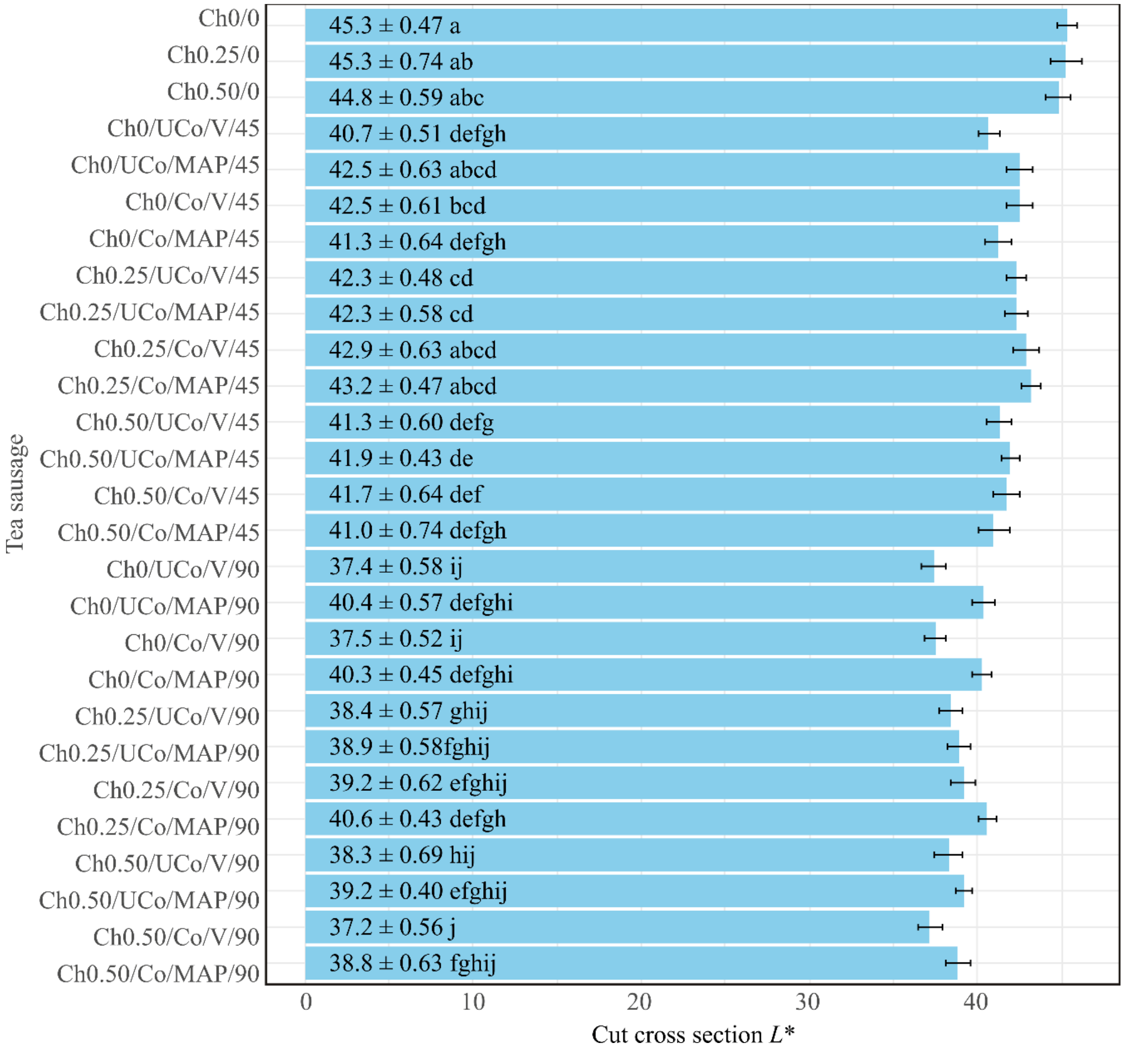

Figure S8. The effect of amount of chitosan, chitosan coating, packaging and storage time on cut cross section *L** values (mean ± SE) of tea sausages. [abcdefghij] $p < 0.05$.

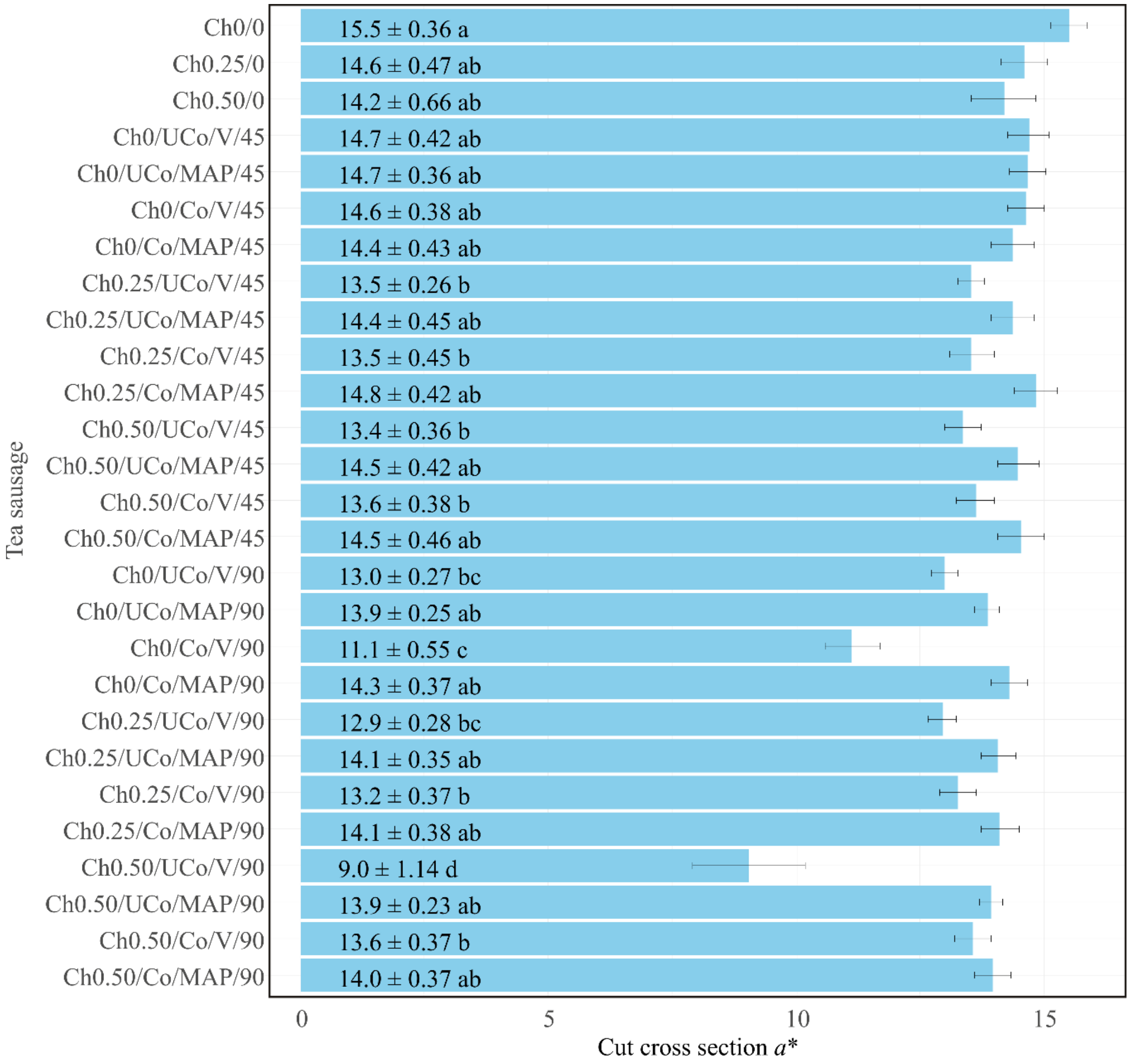

Figure S9. The effect of amount of chitosan, chitosan coating, packaging and storage time on cut cross section a^* values (mean ± SE) of tea sausages.
[abcd] $p < 0.05$.

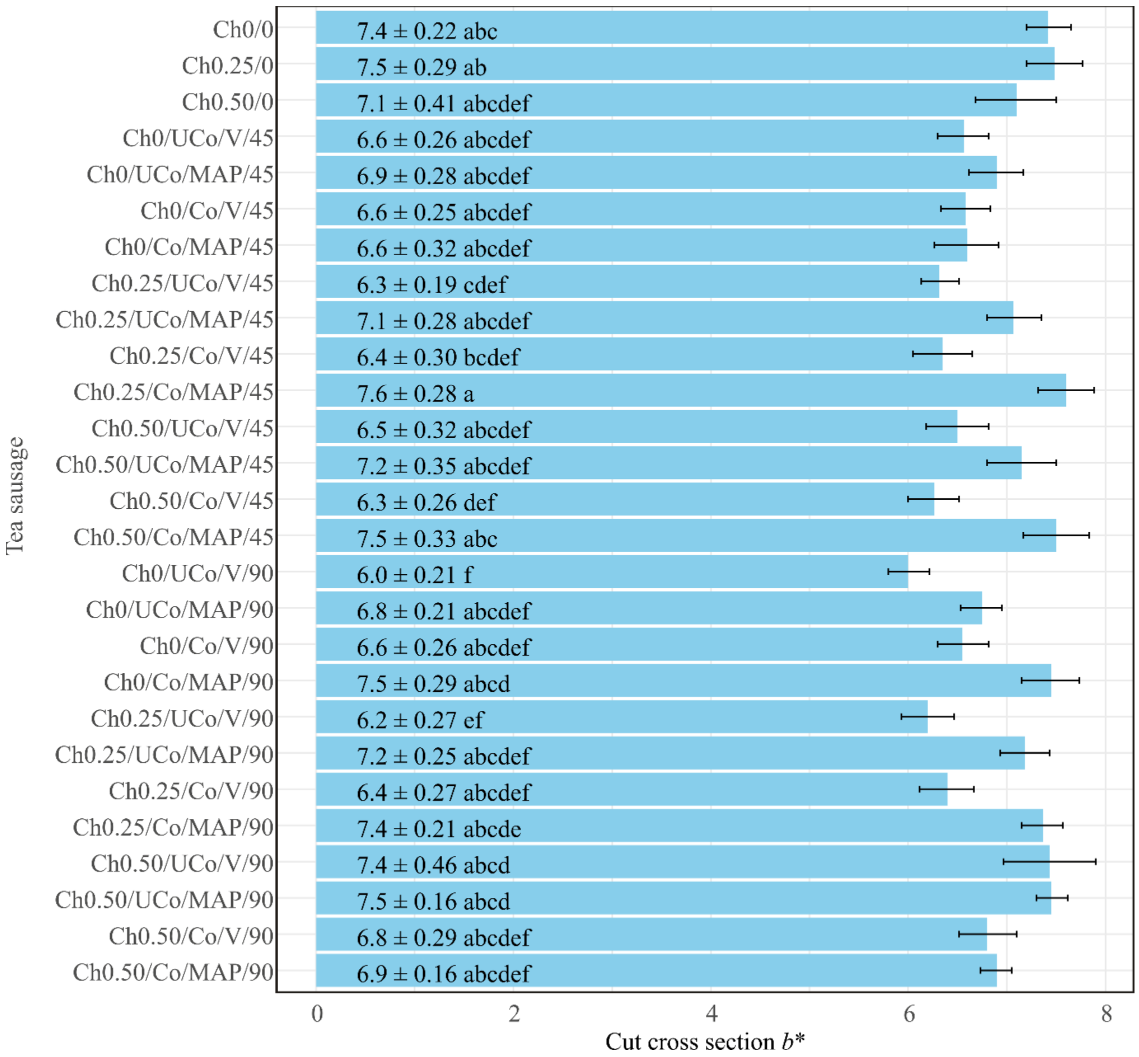

Figure S10. The effect of amount of chitosan, chitosan coating, packaging and storage time on cut cross section b^* values (mean ± SE) of tea sausages. [abcdef] $p < 0.05$.

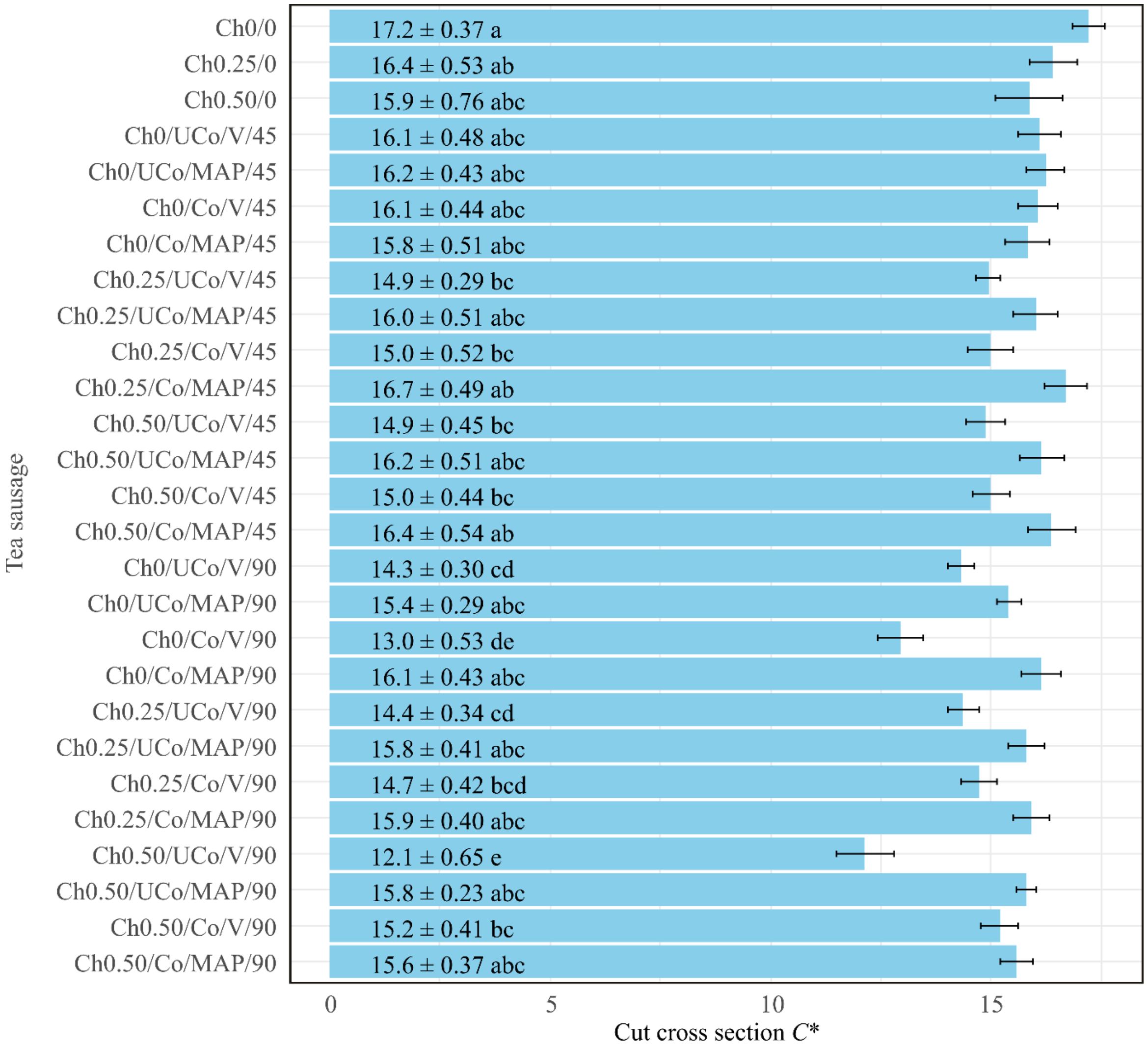

Figure S11. The effect of amount of chitosan, chitosan coating, packaging and storage time on cut cross section C^* values (mean ± SE) of tea sausages. ^{abcde} $p < 0.05$.

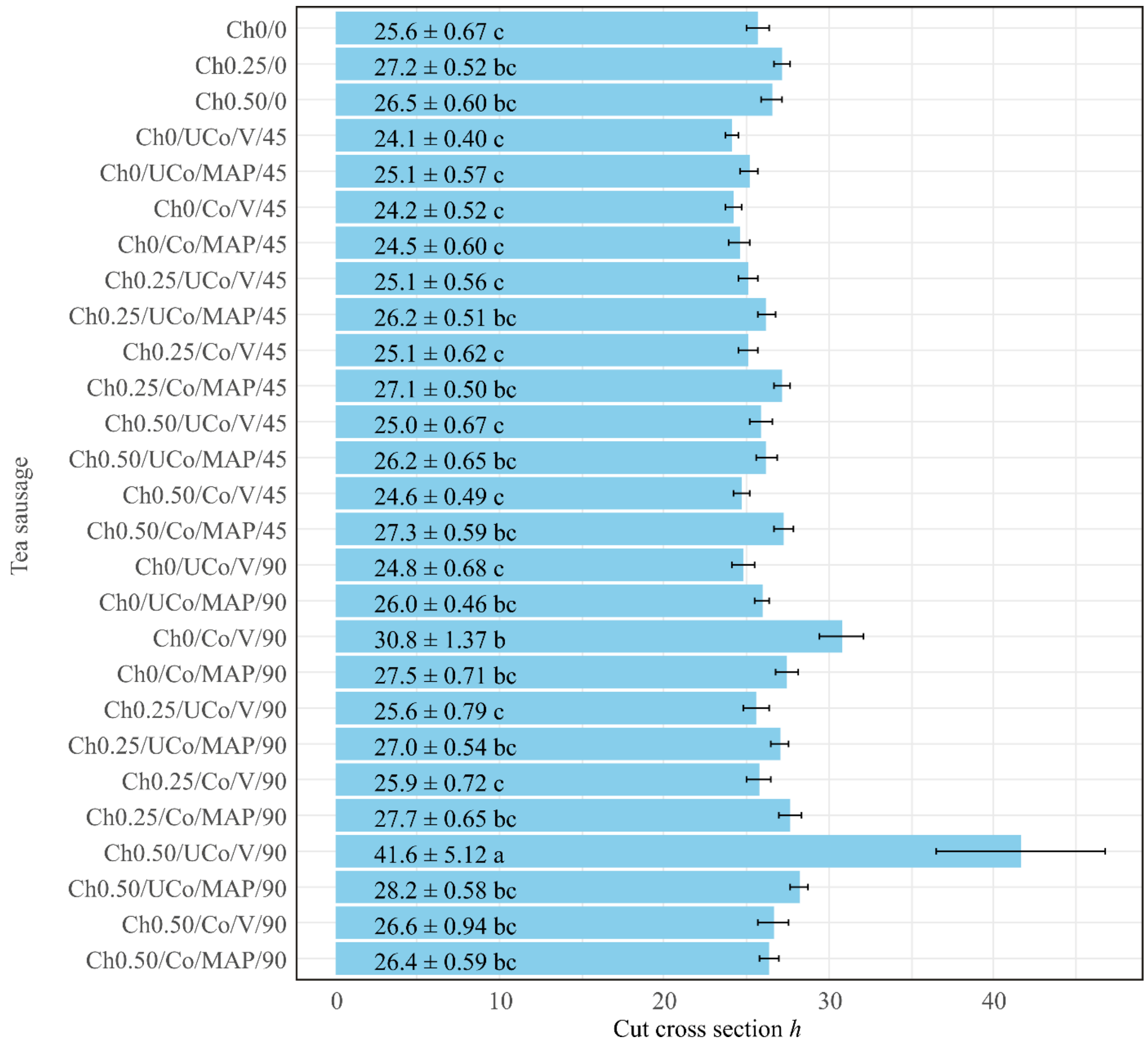

Figure S12. The effect of amount of chitosan, chitosan coating, packaging and storage time on cut cross section *h* values (mean ± SE) of tea sausages. abc $p < 0.05$.

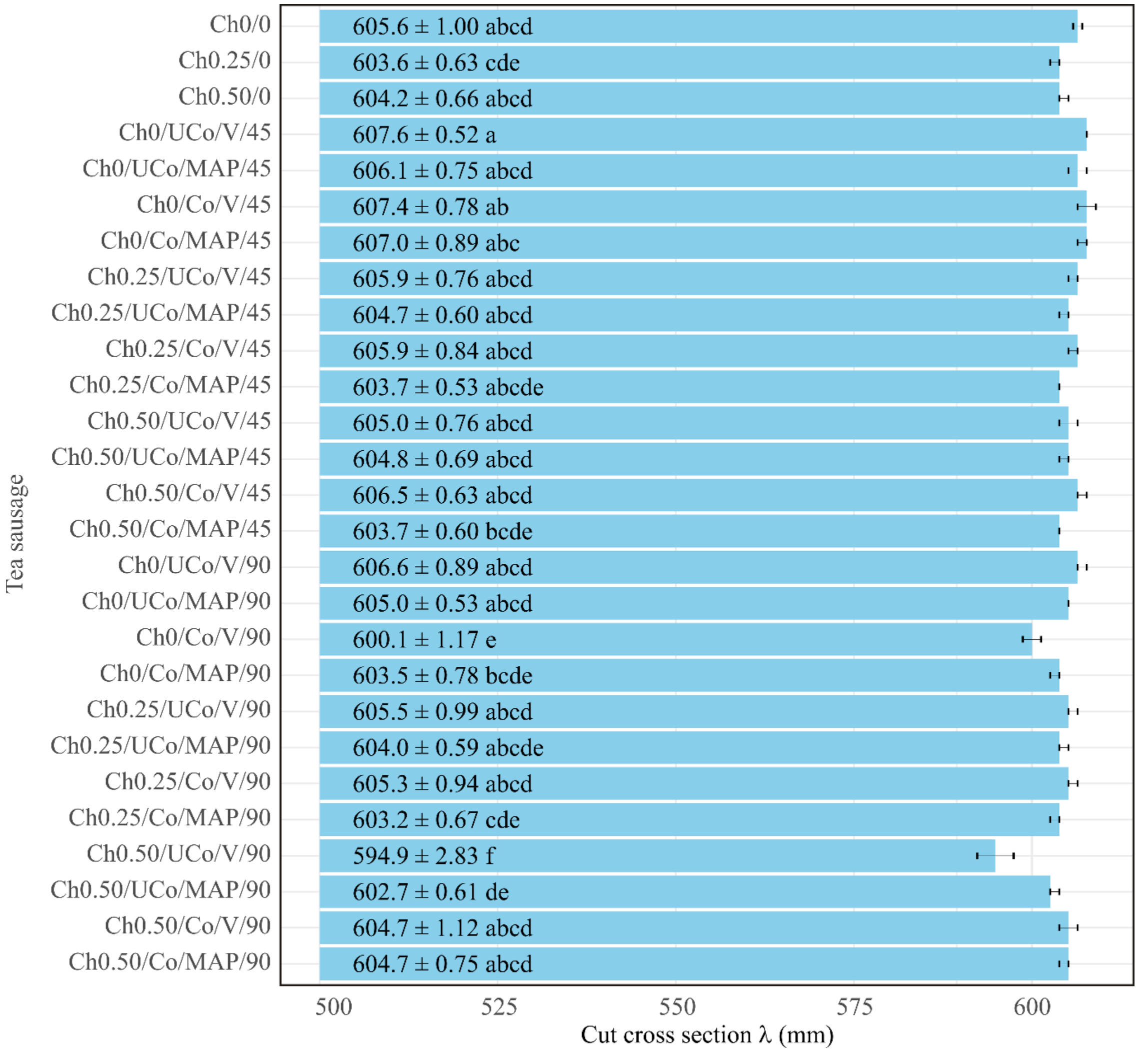

Figure S13. The effect of amount of chitosan, chitosan coating, packaging and storage time on cut cross section λ values (mean ± SE) of tea sausages. [abcdef] $p < 0.05$.

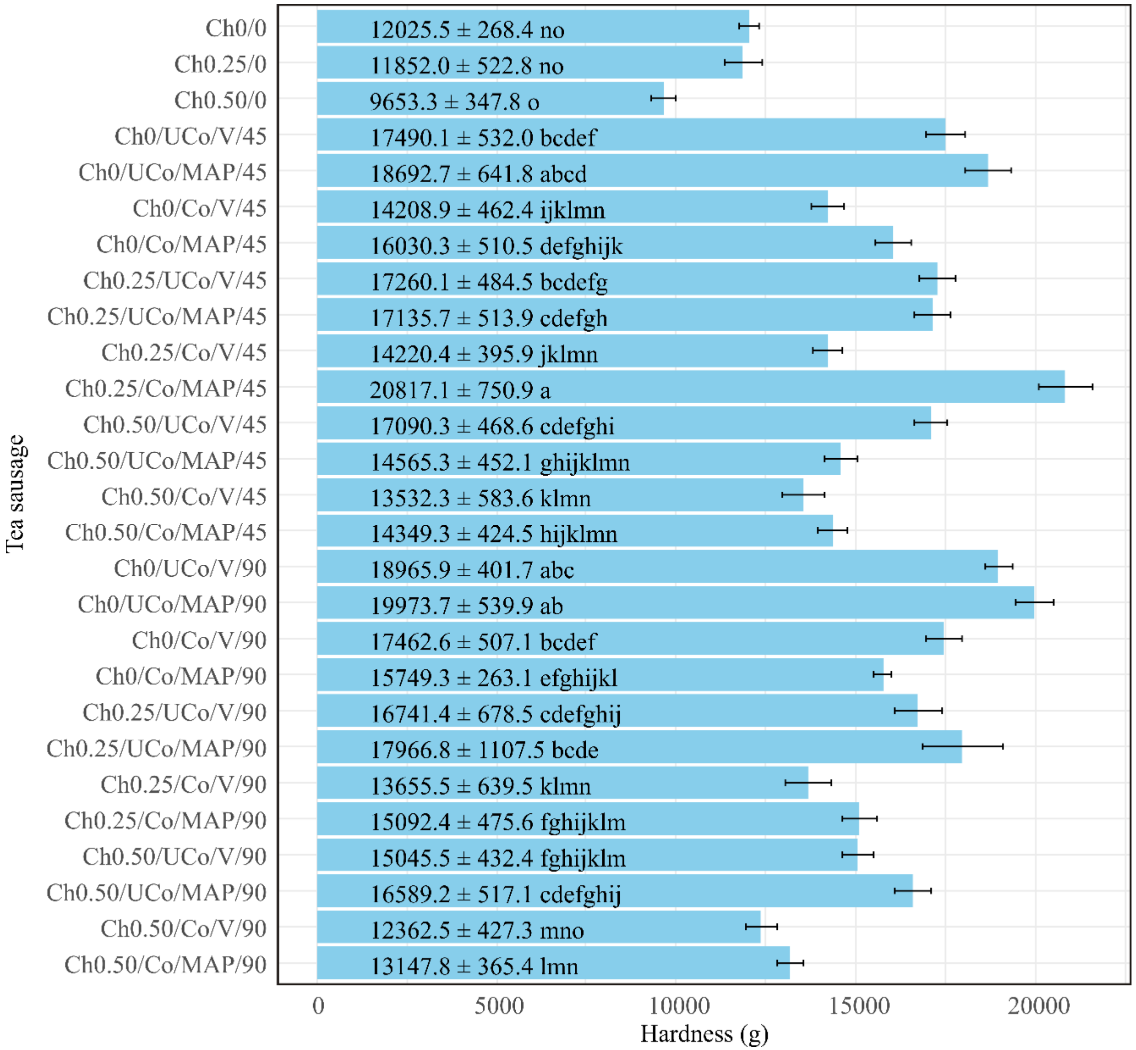

Figure S14. The effect of amount of chitosan, chitosan coating, packaging and storage time on hardness (g) values (mean ± SE) of tea sausages. [abcdefghijklmno] $p < 0.05$.

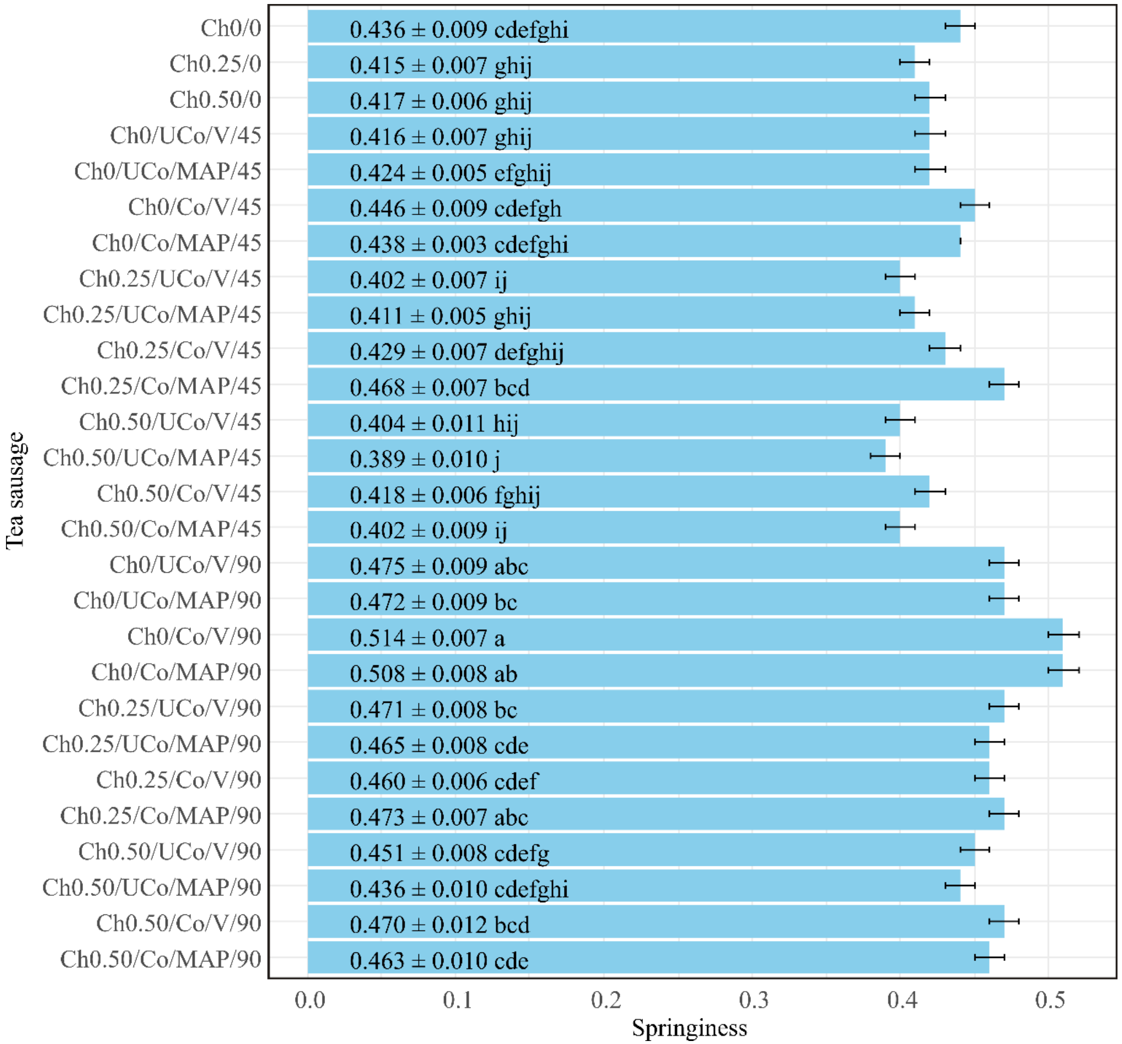

Figure S15. The effect of amount of chitosan, chitosan coating, packaging and storage time on springiness values (mean ± SE) of tea sausages. [abcdefghij] $p < 0.05$.

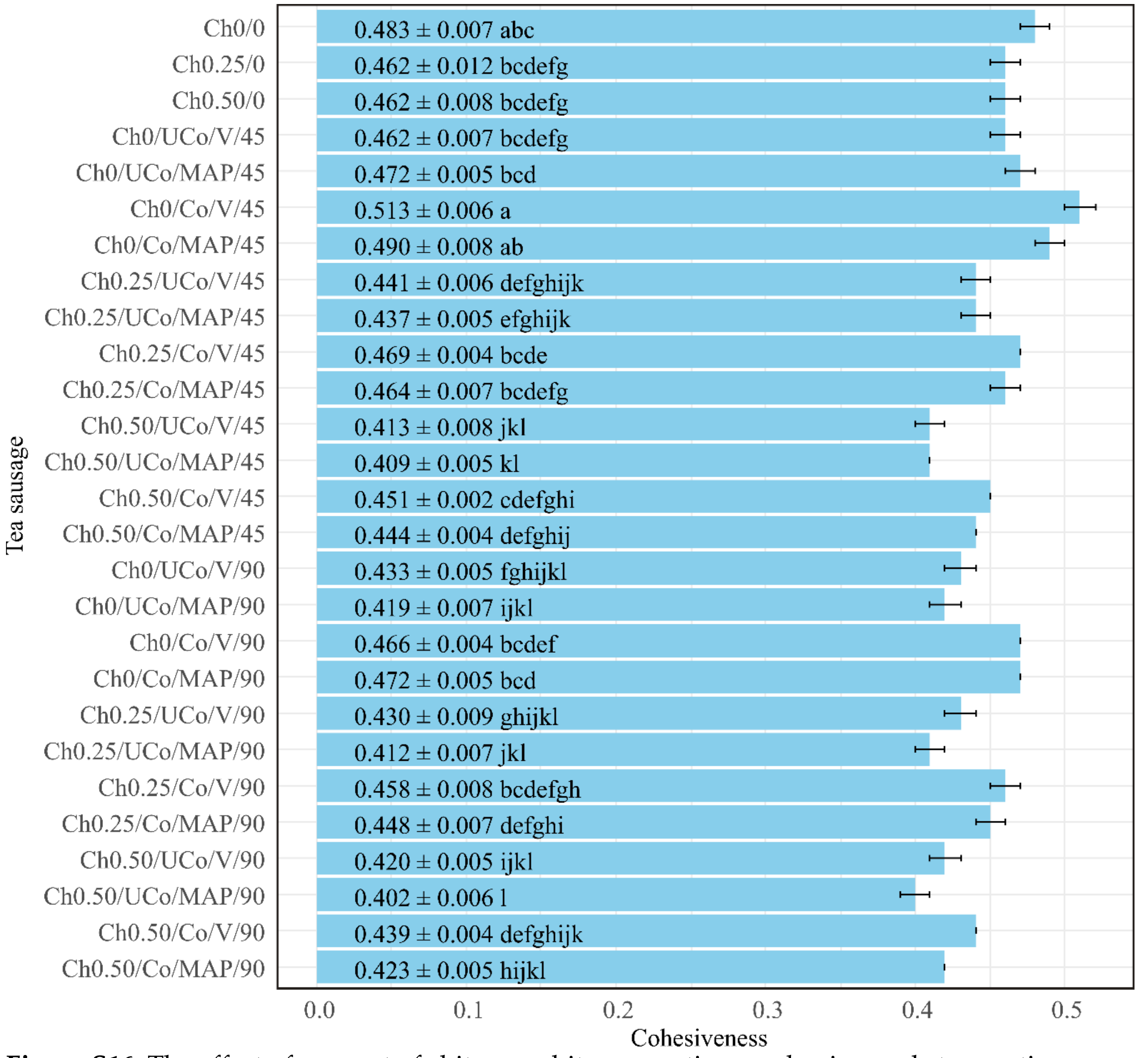

Figure S16. The effect of amount of chitosan, chitosan coating, packaging and storage time on cohesiveness values (mean ± SE) of tea sausages. [abcdefghijkl] $p < 0.05$.

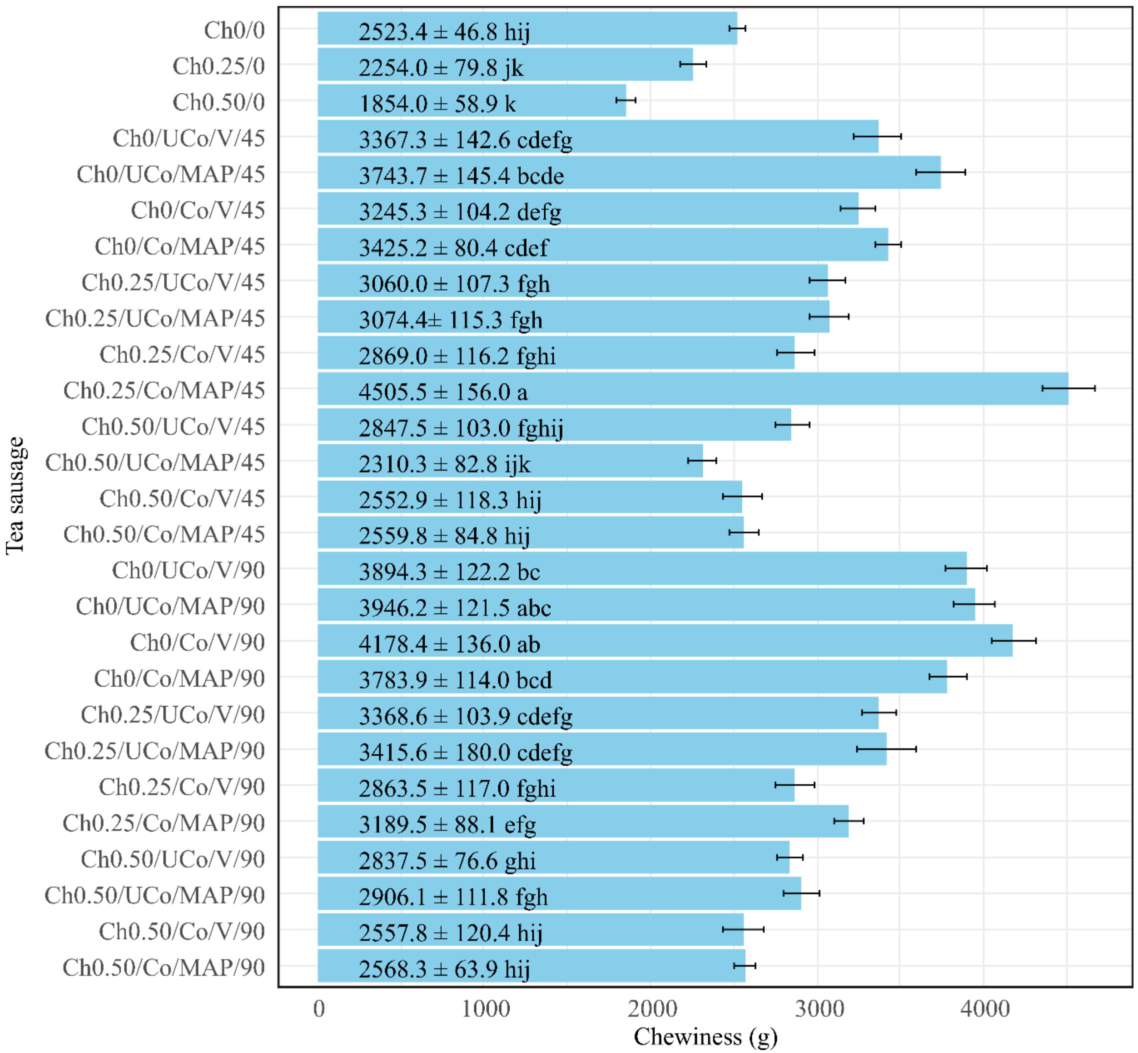

Figure S17. The effect of amount of chitosan, chitosan coating, packaging and storage time on chewiness (g) values (mean ± SE) of tea sausages. abcdefghijk $p < 0.05$.

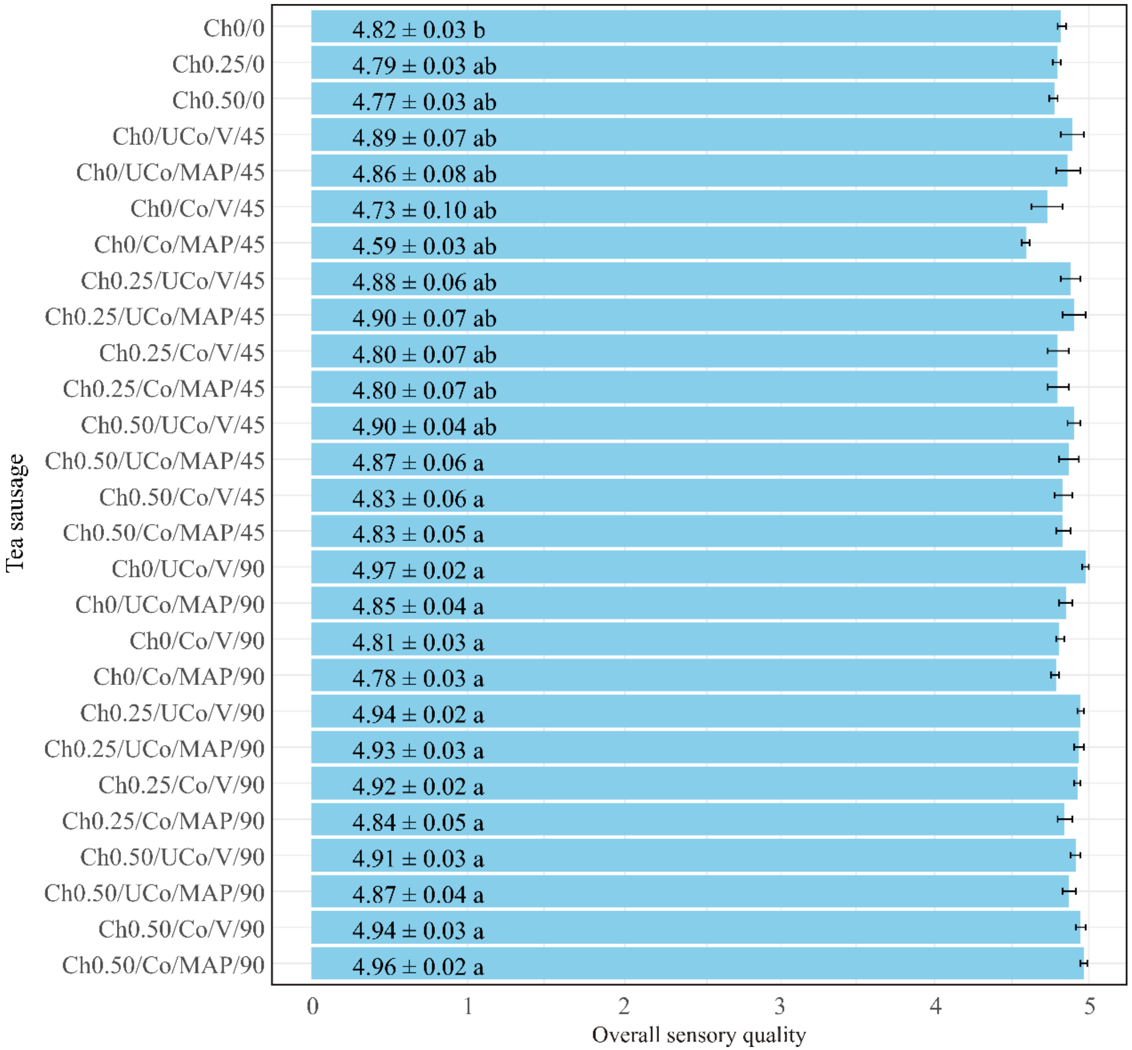

Figure S18. The effect of amount of chitosan, chitosan coating, packaging and storage time on overall sensory quality values (mean ± SE) of tea sausages.
[ab] $p < 0.05$.

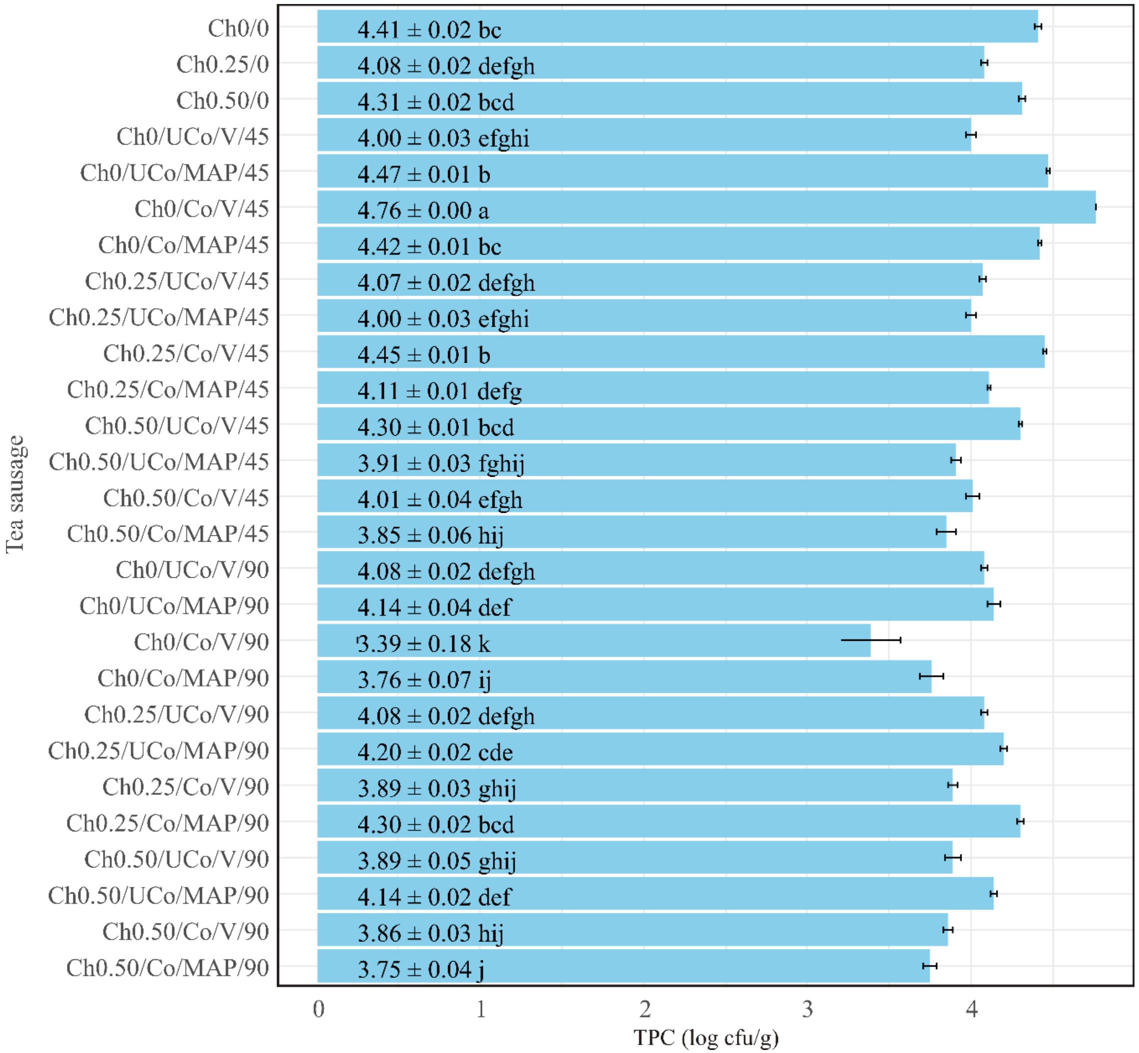

Figure S19. The effect of amount of chitosan, chitosan coating, packaging and storage time on TPC (log cfu/g) values (mean ± SE) of tea sausages. TPC – total plate count; CFU – colony forming units; [abcdefghijk] $p < 0.05$.

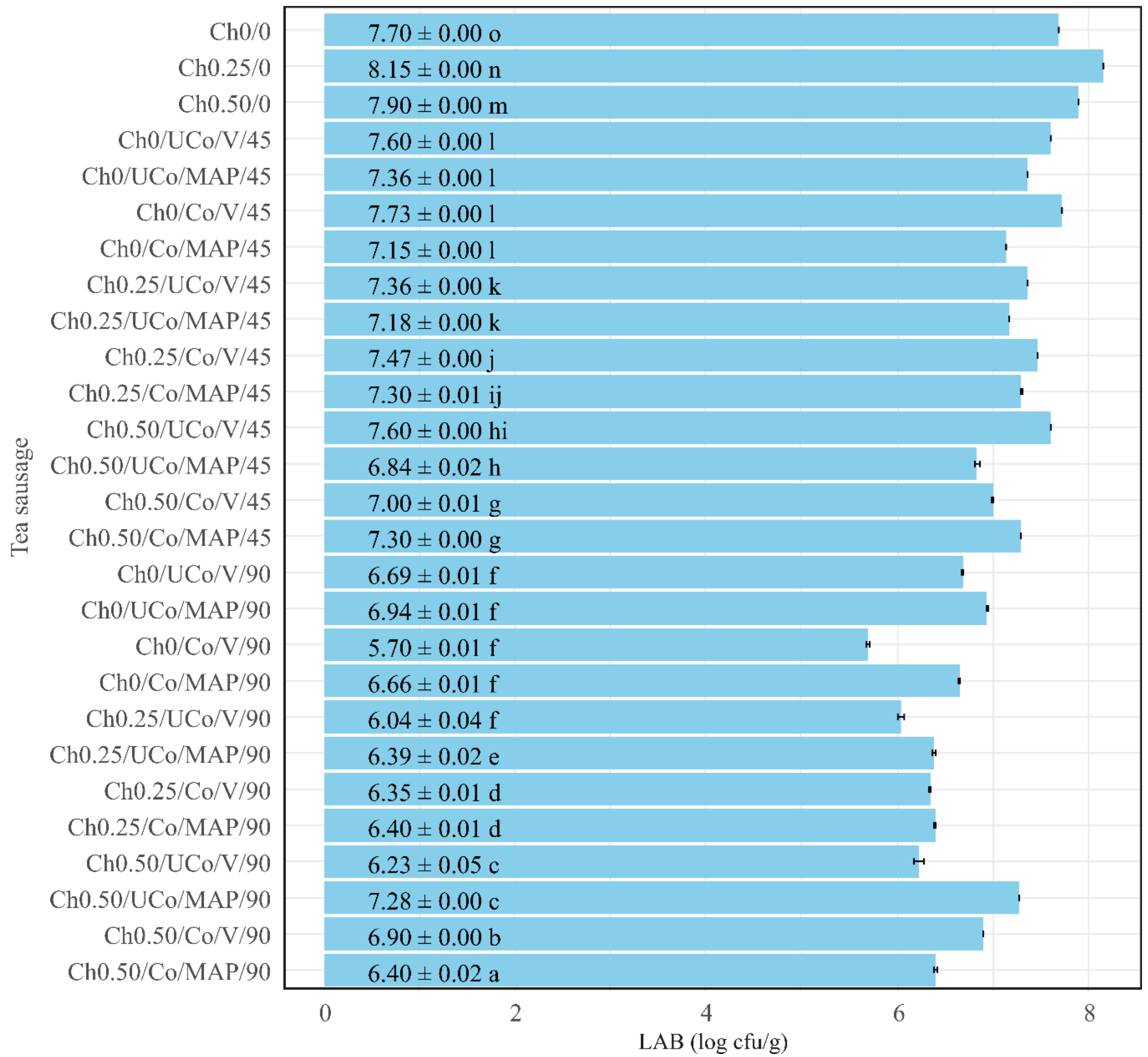

Figure S20. The effect of amount of chitosan, chitosan coating, packaging and storage time on LAB (log cfu/g) values (mean ± SE) of tea sausages. LAB – lactic acid bacteria; CFU – colony forming units; [abcdefghijklmno] $p < 0.05$.
